# 3D Photothermal Cryogels for Solar-Driven Desalination

**DOI:** 10.1021/acsami.1c05087

**Published:** 2021-06-22

**Authors:** Siew-Leng Loo, Lía Vásquez, Muhammad Zahid, Federica Costantino, Athanassia Athanassiou, Despina Fragouli

**Affiliations:** † Smart Materials, Istituto Italiano di Tecnologia, Via Morego 30, 16163 Genova, Italy; ‡ Dipartimento di Chimica e Chimica Industriale (DCCI) Università degli Studi di Genova, Via Dodecaneso 31, 16146 Genova, Italy; § Interdisciplinary Laboratories for Advanced Materials Physics (i-LAMP) and Dipartimento di Matematica e Fisica, Università Cattolica del Sacro Cuore, Via Musei 41, 25121 Brescia, Italy

**Keywords:** interfacial solar evaporation, solar−thermal conversion, porous hydrogels, polypyrrole, poly(sodium acrylate)

## Abstract

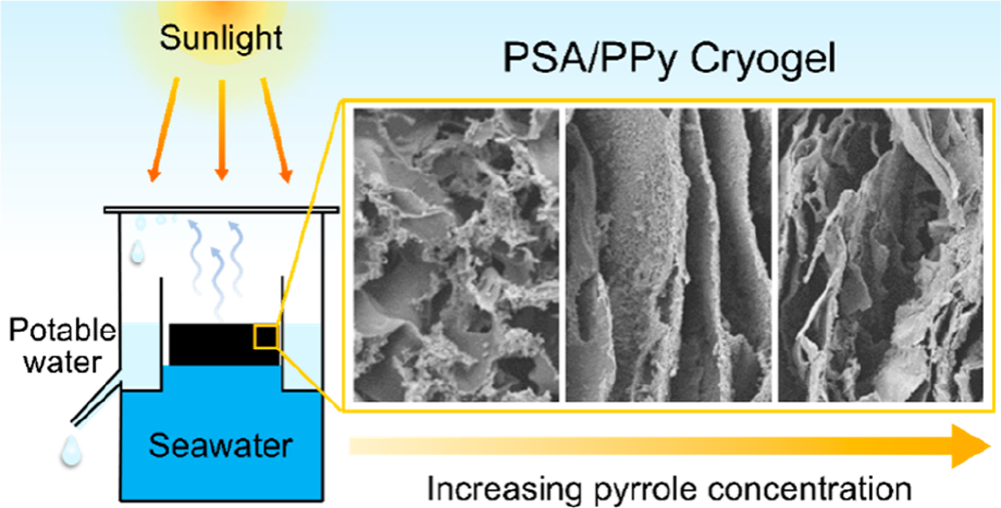

This paper reports the fabrication of photothermal cryogels for freshwater production *via* the solar-driven evaporation of seawater. Photothermal cryogels were prepared *viain situ* oxidative polymerization of pyrrole with ammonium persulfate on preformed poly(sodium acrylate) (PSA) cryogels. We found that the pyrrole concentration used in the fabrication process has a significant effect on the final PSA/PPy cryogels (PPCs), causing the as-formed polypyrrole (PPy) layer on the PPC to evolve from nanoparticles to lamellar sheets and to consolidated thin films. PPC fabricated using the lowest pyrrole concentration (i.e., PPC10) displays the best solar-evaporation efficiency compared to the other samples, which is further improved by switching the operative mode from floating to standing. Specifically, in the latter case, the apparent solar evaporation rate and solar-to-vapor conversion efficiency reach 1.41 kg m^–2^ h^–1^ and 96.9%, respectively, due to the contribution of evaporation from the exposed lateral surfaces. The distillate obtained from the condensed vapor, generated *via* solar evaporation of a synthetic seawater through PPC10, shows an at least 99.99% reduction of Na while all the other elements are reduced to a subppm level. We attribute the superior solar evaporation and desalination performance of PPC10 to its (i) higher photoabsorption efficiency, (ii) higher heat localization effect, (iii) open porous structure that facilitates vapor removal, (iv) rough pore surface that increases the surface area for light absorption and water evaporation, and (v) higher water-absorption capacity to ensure efficient water replenishment to the evaporative sites. It is anticipated that the gained know-how from this study would offer insightful guidelines to better designs of polymer-based 3D photothermal materials for solar evaporation as well as for other emerging solar-related applications.

## 1 Introduction

Although water is critical in sustaining global health as well as socio-economic development of the modern society, providing fresh water universally in a sustainable manner is a challenging task. Global water scarcity is expected to worsen as the depletion of freshwater sources, resulting from saline intrusion and anthropogenic contamination, is further exacerbated by the climate change and the escalating water demand due to rapid population growth.^1−3^ Addressing the issue of global water security is therefore urgent, particularly for the poor and vulnerable populations in disaster-prone regions, as they are most afflicted by water scarcity due to the lack of access to conventional infrastructure and power sources to produce clean water.^4,5^ In congruence with the vision of the Sustainable Development Goals of the United Nations, providing universal access to water calls for resilient and adaptive solutions that can produce water *via* simple approaches at a low cost, minimizing at the same time, chemical consumption and its environmental impacts.^4^

As seawater and saline aquifers account for 97.5% of all water on Earth, desalting even a small fraction of this abundant resource can greatly contribute to securing adequate global freshwater supply for the coming decades.^6^ Desalination technologies based on solar-driven evaporation, in particular, can be a potential solution that meets the aforementioned criteria as solar energy is clean, renewable, and a sustainable alternative to fossil fuels. Specifically, this technique involves the absorption and conversion of solar energy into heat to drive the evaporation of water from nonvolatile contaminated solutions, followed by the collection of the purified water *via* vapor condensation. Compared to the traditional solar-driven evaporation that involves the bulk heating of water, the interfacial solar evaporation process is more energy-efficient as it confines the photoconverted heat to the evaporative portion of water *via* the heat localization effect presented by well-designed photothermal materials.^7,8^ Compared to other mainstream desalination technologies (e.g., reverse osmosis and multistage flash)^9−12^ that require high-energy inputs, sophisticated operational controls as well as large-footprint investments, interfacial solar-driven evaporation is an infrastructure-independent technique that can be operated remotely without additional power supply.^13,14^ Furthermore, it can be used to desalinate hypersaline waters (up to 100 g of dissolved salt per kg of seawater, i.e., equivalent to the water of the Dead Sea) and produce a distillate of superior quality.^15,16^ Besides seawater desalination, solar-driven evaporation has been also shown capable of purifying industrial wastewaters contaminated by strong acids and bases, heavy metals, nonvolatile organics (dyes and detergents), and even radioactive wastes.^16−19^

As interfacial solar evaporation is a coupled process of solar-energy absorption, solar-to-heat (photothermal) conversion, heat transfer, and water transport, efficient solar evaporators typically must show (i) strong broadband light absorption, (ii) high photothermal conversion efficiency, (iii) effective heat localization with minimal heat loss, (iv) sustainable and rapid water supply to the evaporative surface, and (v) efficient vapor escape.^8^ Therefore, the first important criterion for an efficient solar evaporator is to be able to absorb and convert solar energy into heat. On the basis of this criterion, various solar absorbers such as plasmonic nanoparticles,^20^ semiconductors,^21^ metal–organic frameworks,^22^ mXene,^23^ graphene/graphite,^24^ and carbon nanotubes^25^ have been explored as photothermal fillers in solar evaporators. However, the aforementioned solar absorbers tend to have high thermal-conductivity that may contribute to significant heat loss, resulting in inferior vapor-generation efficiency.^7^ On the contrary, conjugated polymers such as polypyrrole (PPy), polydopamine, and polyaniline among others are also very efficient for photothermal conversion due to their strong near-infrared (NIR) light absorption and their ability to convert incident photons into heat *via* nonradiative relaxation and molecular vibrations in their chains.^26^ Specifically, owing to its high *in vivo* biocompatibility and low biotoxicity, PPy has been extensively studied as a photothermal agent for cancer therapy and, more recently, in the field of solar evaporation.^27,28^ Compared to other solar absorbers, PPy offers the advantages of (i) lower thermal-conductivity that can minimize heat loss and (ii) facile chemical polymerization method that can be easily scaled up.

Most solar absorbers need to be combined with a support matrix in order to arrive to a robust, practical, and functional solar evaporator. Compared to two-dimensional (2D) solar evaporators in the form of membranes, three-dimensional (3D) porous components endow the final system with enhanced solar evaporation performance due to their (i) superior light-trapping efficiency, (ii) efficient water-transport properties, (iii) presence of unhindered vapor escape channels, and (iv) increased surface area for evaporation.^29−31^ Macroporous hydrogels, in particular, have been shown to be a suitable platform for constructing 3D solar evaporators due to their (i) facile fabrication with tailorable pore structures,^29,32,33^ (ii) hydrophilicity and capillarity that facilitate water transport,^34,35^ and (iii) high water-content that can prevent the supersaturation of salt on their surface.^36^

It can be, therefore, expected that the combination of PPy with a 3D macroporous hydrogel substrate would lead to a solar evaporator with enhanced performance. To test this hypothesis, a 3D solar evaporator is formed using a poly(sodium acrylate) (PSA) cryogel as a substrate to be functionalized with PPy *viain situ* oxidative polymerization by ammonium persulfate (APS). So far, PSA cryogels—which have been shown to have high elasticity and rapid water-absorption property—have been functionalized with silver nanoparticles and nanozerovalent iron, and demonstrated remarkable performance when applied for point-of-use water disinfection, filtration, and adsorptive removal of pollutants.^37−40^ The high charge-density of PSA cryogels, due to the presence of numerous anionic carboxylate groups, is anticipated to facilitate the *in situ* polymerization of PPy as it not only improves the interaction with the pyrrole precursor but also acts as a dopant that stabilizes the cationic radicals during the polymerization process.^41^ In addition, compared to other support materials for PPy-based solar evaporators, PSA cryogels present macroporous structures that can facilitate rapid water transport^37,38,40^ and efficient vapor escape promoting a high solar evaporation efficiency. Furthermore, in combination with the high water-content in the PSA network, salt clogging may be more efficiently prevented due to the reduction of the local salt concentration at the evaporating surface.^42−44^

As shown herein, PPy-coated PSA cryogels (PPCs) fabricated from different pyrrole concentrations result in 3D hybrids with varying morphologies, water absorption, and photothermal properties. In-depth discussions regarding the mechanism of PPy formation within the PSA framework, and the effects of the PPy layer on the pore structures and morphologies of the resultant composites are provided. Furthermore, we have investigated the relationship between the different pore structures and morphologies of the PPC samples with their functions, necessary for an effective photothermal performance (e.g., water transport, light-harvesting efficiency, heat localization effect, vapor escape efficiency, solar evaporation efficiency, and salt-resistance). We anticipate that the insightful discussion highlighted in this work can advance the field of solar desalination, as well as other emerging solar-related applications, as it provides pragmatic guidelines that would lead to an improved design and fabrication of (polymer-based) photothermal materials with tailored properties for enhanced performance.

## 2 Experimental Section

### 2.1 Fabrication of Photothermal Cryogels

For the design principles and the synthesis of the PSA cryogels, we have adopted a previously reported method^40^ with slight modifications. Briefly, ammonium persulfate (APS, 98% purity, Sigma-Aldrich) and *N*,*N*,*N*,*N*′-tetramethylethylenediamine (TEMED, ≥99%, Sigma-Aldrich) were added to a prechilled reaction mixture, containing sodium acrylate (SA, 97%, Sigma-Aldrich) and *N*,*N*′-methylenebis(acrylamide) (MBA, 99%, Sigma-Aldrich). The APS and TEMED concentrations in the final reaction mixture were 3.50 mM and 0.125% (v/v), respectively, while the monomer concentration (SA + MBA) used was 12% (w/v) at a cross-linker ratio of 0.05 (mol of MBA/mol of SA). A fixed volume of the resultant reaction mixture was transferred into a Petri dish that was then placed in a freezer (−25 ± 2 °C) for 24 h to ensure complete polymerization. After removal from the freezer, the resultant PSA cryogels (while still in the frozen state) were cut into small disks by the means of a punch stamp. The cryogel disks were then rinsed with Milli-Q water (18.2 MΩ cm at 25 °C) before they were left to dry in a freeze-dryer (CHRIST Epsilon 2–4 LSCplus, −45 °C). To fabricate PPy-coated PSA cryogels (PPCs), slices of the dry PSA cryogel disks (0.1 g) were immersed in a 50 mL aqueous solution of pyrrole of varying concentrations (i.e., 10, 20, and 30% v/v) for 24 h under rigorous vortexing (800 rpm). After that, the excess pyrrole solution was drained. Then, the pyrrole-coated PSA cryogels were immersed in 50 mL of APS solutions of varying concentrations (i.e., 0.5, 0.1, and 1.5 M) for another 24 h to ensure complete PPy polymerization. Note that the molar ratio of APS to pyrrole in the two solutions, prior to the dipping process, was kept constant in all cases. The resultant PPC samples were first cleaned by removing the excess solution, both inside and outside the gel, *via* vacuum filtration followed by rehydration in Milli-Q water. The filtration-and-rehydration process was repeated until the filtrate became clear. Subsequently, the PPCs were further cleaned by immersion in Milli-Q water under vortex (800 rpm) overnight. Then, the excess solution was drained and the cryogel was reimmersed in Milli-Q water and mixed under vortex overnight. This process was repeated until the excess solution drained out became clear. Then, the PPCs were left to dry in a freeze-dryer. For reference purposes, a PPy film was also prepared by polymerizing a 10% pyrrole solution in the presence of 0.5 M APS. After sonication for 1 h, the mixture was poured into a Teflon dish to complete the polymerization reaction. After 24 h, the mixture was left to dry in an oven at 60 °C. The stability of the PPy incorporated into PSA cryogels was confirmed by analyzing the total organic carbon (TOC) concentration, as determined using a uniTOC lab analyzer (membraPure), in the Milli-Q water in contact with PPC for 1 h with or without 1 Sun irradiation. In both cases, the TOC concentration in the leachate was negligible (<1 ppm), indicating the stability of the PPy layer on the PSA.

### 2.2 Materials Characterization

The morphology of the foams was studied using a scanning electron microscope (SEM) (JEOL JSM-6490LA) at an acceleration voltage of 10 kV. To do so, the samples were coated with a layer of 10 nm Au. Energy-dispersive X-ray spectrometry (EDS) analyses were carried out using a JEOL JSM-6490LA SEM equipped with a tungsten filament (W) working at a 15 kV accelerating voltage. Nitrogen adsorption–desorption isotherms were performed by an Autosorb-iQ gas sorption analyzer (Quantachrome Instruments). The specific surface area and the pore size distribution were determined by employing the Brunauer–Emmett–Teller (BET) and Barrett–Joyner–Halenda (BJH) techniques. The real density of the cryogels was determined by the means of a pycnometer (Pycnomatic ATC). The samples were placed in 4 cm^3^ cuvette and suspended in the pycnometer at 20.00 ± 0.01 °C with helium as the measuring gas. Ten measurements were performed for each sample with an accuracy of ±0.06%. The skeletal density obtained was then used to calculate the effective porosity and pore-size distribution in the macropore range using Pascal 140 Evo and Pascal 240 Evo mercury-intrusion porosimeters (MIP; Thermo Fisher Scientific), which have mercury-intrusion pressure ranges of 0.001–0.4 and 0.1–200 MPa, respectively. The data obtained from both porosimeters were combined and correlate to an equivalent pore-size range of 0.01–1000 μm by the Washburn’s equation using the SOL.I.D Evo Software, assuming a mercury contact angle of 140° and mercury surface tension of 0.48 N m^–1^. The MIP pore surface area was estimated using a cylindrical and plate surface area model. The chemical composition and the possible chemical interactions of the components of the samples were studied using a Fourier transform infrared (FTIR) spectrometer (Vertex 70v FT-IR, Bruker) coupled to a single-reflection attenuated total reflection (ATR) accessory (MIRacle ATR, PIKE Technologies). All spectra shown were averaged from 128 repetitive scans recorded in the range from 3800 to 600 cm^–1^ with a resolution of 4 cm^–1^. Thermal images were recorded using an infrared camera (IR-camera) FLIR X6580sc and subsequently analyzed using ResearchIR software. The thermal decomposition profiles and the first derivative curves were obtained through thermogravimetric analyses (TGA) using a Q500 system (TA Instruments) with a heating rate of 10 °C min^–1^ from 30 to 800 °C in a N_2_ atmosphere. The water contact angle was measured using a DataPhysics OCA 20 contact angle goniometer. Thermal conductivity measurements of dry samples were conducted using a TCi thermal conductivity analyzer. The optical transmittance and reflectance spectra of the samples were recorded in the range 200–2500 nm by an UV–vis–NIR spectrophotometer Cary 5000 equipped with an integrating sphere and calibrated with an MgO mirror before starting the measurements. The photoabsorption efficiency was calculated according to
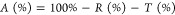
1where *R* and *T* are the percentages of reflectance and transmittance of the sample, respectively. The percentages of reflectance and transmittance were determined *via* integrating the area under spectrum.

### 2.3 Water-Absorption Studies

The water-absorption properties of the samples were determined by gravimetric analysis. For the determination of the swelling profile, for each sample, a gel disk with ca. 9 mm diameter and 2 mm height was allowed to swell in an excess of Milli-Q water or synthetic seawater (3.5 wt %). Water uptake by the gel sample was determined by measuring the cumulative mass increase at a predetermined time interval. Excess surface water was gently wiped off using a damp paper towel before measuring the mass of the swollen gel. The water absorption at time *t* was calculated as follows:
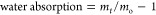
2where *m*_*t*_ and *m*_o_ are the masses of the swollen gel at time *t* and dried gel, respectively. The volumetric swelling degree was determined by taking the ratio of the volume of the swollen gel to that of the xerogel. The water-absorption capacity of the samples is the water absorption at their equilibrium swollen state. To determine the water-vapor sorption under a saturated condition, we equilibrated dry samples in a chamber saturated with water vapor (but not in contact with liquid water) until they reached constant mass.

### 2.4 Solar Evaporation and Desalination Experiments

Solar evaporation tests were conducted at an ambient temperature of 19 ± 1 °C and a relative humidity (RH) of 60 ± 5%. The solar simulator used was a ScienceTech SLB-150B (Class BAA) with an AM1.5G air mass filter and calibrated by Oriel reference solar cell and meter (91150 V). Unless otherwise specified, the solar evaporation tests were conducted by placing a swollen cryogel disk on the surface of water. It should be mentioned that the samples have a self-floating ability, thus no additional devices or support materials were added. The entire surface area of the water was ensured to be covered by the sample. The mass loss of water over time under 1 Sun irradiation was measured using an analytical balance (Kern, 0.01 mg accuracy). The final evaporation rates, *ṁ*, reported are the result of the subtraction of the evaporation rate of the PPC samples in the dark from the measured evaporation rates under 1 Sun illumination.^45,46^ Solar-to-vapor conversion efficiency, η, was calculated using eq 3:^7^
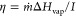
3where *I* is the power density of the incident light (1 kW m^–2^) and *ΔH*_vap_ is the evaporation enthalpy of water, which was calculated as follows:
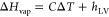
4where *C* is the specific heat capacity of water (4.2 kJ °C^1–^ kg^–1^), *ΔT* represents the temperature increase of water during vaporization, and *h*_LV_ is the latent heat of vaporization of water (2436 J g^–1^ at 27 °C).^47^ Desalination experiments were conducted in a condensation chamber (to facilitate collection of condensed vapor), as shown in Figure S1 in the Supporting Information, using synthetic seawater samples (3.5 wt %) prepared by dissolving sea salts (NutriSelect Basic, S9883) in deionized water. Table S1 in the Supporting Information summarizes the composition of the as-prepared synthetic seawater. The concentrations of Na, K, Ca, Mg, B, and Sr in the distillate were analyzed using an inductively coupled plasma optical emission spectrometer (ICP-OES) (iCAP 6300, ThermoScientific); samples were acidified with HNO_3_ prior to analysis.

## 3 Results and Discussion

### 3.1 Fabrication and Morphology of Photothermal Cryogels

**Figure 1:**
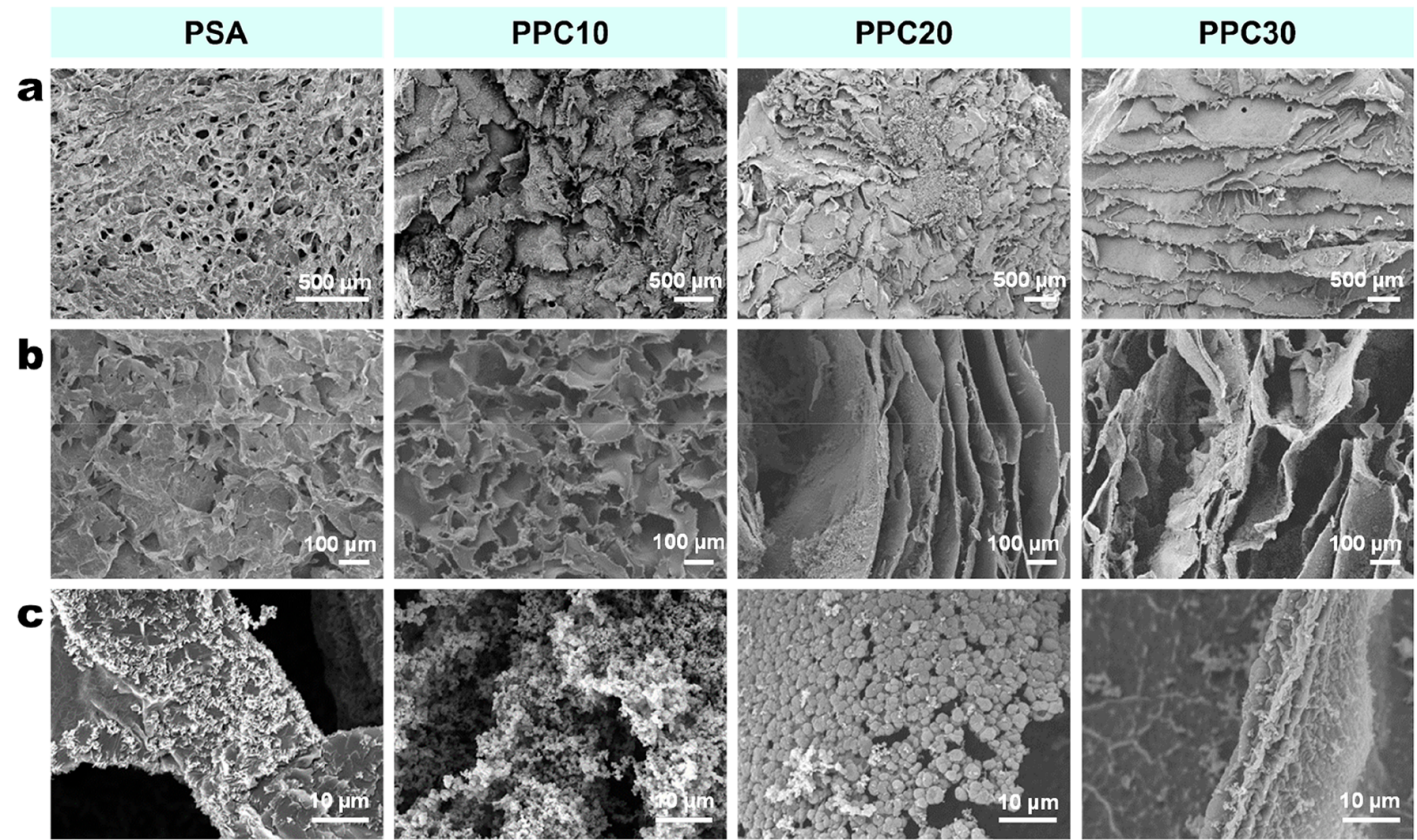
SEM images showing (a) the top surface and (b) the cross-sectional morphology of the samples. (c) High-magnification SEM images elucidating the detailed morphology of the samples.

PPC samples were prepared *viain situ* oxidative polymerization of pyrrole using APS as the oxidant and PSA as the substrate. Pyrrole is oxidized by APS to form reactive pyrrole cation radicals, which can then react with the surrounding pyrrole molecules to form long PPy chains.^48,49^ The presence of the abundant anionic charges on PSA, in particular, contribute to the stabilization of the pyrrole radical cations and act as a charge-balancing dopant to the PPy formed.^41,49^ As the PPy polymerization takes place, the cryogels progressively darken and eventually they become black (Figure S2). Hereafter, the PPC samples will be referred to as PPC*x* where *x* refers to the precursor pyrrole concentration in the dipping solution used for the synthesis (i.e., 10, 20, or 30% v/v). As shown in Figure 1a, a significant modification of the top surface morphology of the PSA sample occurs in the presence of PPy, and this becomes more evident as the pyrrole concentration increases. The morphological transformation of the samples in the presence of PPy is even more evident in the cross-section analysis of all samples (Figure 1b). Specifically, the macroporous and honeycomb structures of PSA are retained in PPC10, but a further increase of pyrrole concentration results in the formation of multilamellar structures in the whole volume of the samples. Under a high-magnification examination, it is noted, that, for PPC10, the modification with PPy has transformed the less-rough pore surface of PSA to one that is decorated with clustered florets (Figure 1c, with further details in Figure S3). Specifically, the surface is covered by a loose network of PPy particles (with a mean size of 500 nm), which is a typical morphology observed for PPy formed *via* chemical oxidation using APS.^27,50^ Increasing the pyrrole concentration to 20% results in the stacking of PPy microgranules (mean size of 1.9 ± 0.5 μm) to form lamellar sheets, while, when the pyrrole concentration is further increased to 30%, the PPy granules densely pack, forming highly consolidated PPy films with a smoother surface (Figure 1c).

EDS mapping was used to study the distribution of PPy in the PPC samples. As shown in Figures S4–S6 in the Supporting Information, the EDS spectra of the top and bottom surfaces and the cross-section of PSA were characterized by the presence of C, O, and Na peaks. Although we could not discern the N peak attributed to the presence of PPy due to the similar atomic weights of N with C and O, we could observe the disappearance of the intense Na peak (assigned to PSA) coupled with the appearance of an S peak (due to the remnants of the oxidizing agent used to prepare PPy) (Figure S4–S6). Since the cross-sectional, top, and bottom surfaces of all the PPC samples show the complete disappearance of Na, it is likely that the surface of PSA has been completely covered by PPy for all PPC samples. Furthermore, we note that the signal ratios for C/O in the EDS spectra show a general increase from PSA (i.e., 1.2) to the PPC samples (i.e., 2.5–4.4) (Figures S5 and S6). This provides further support that the PPy (which is less oxygen-rich compared to PSA and consists mainly of C and N) is covering the majority of the PSA surface.

The pore structures of the developed materials were further studied with MIP and N_2_ physisorption analyses, which can provide complementary information regarding the characteristics of the macropores and micro/mesopores such as the specific surface area, pore volume, and the pore-size distribution. MIP analysis allows for the quantification of detailed parameters about the macro-structures (>10 nm) of the materials, while N_2_ physisorption analysis was adopted to investigate the structural characteristics of the mesoscopic (2–50 nm) and microscopic (<2 nm) pores.^51^ The MIP pore-size distribution analysis revealed that the increasing concentration of pyrrole used to prepare the PPCs results in a shift toward a larger modal macropore-size (Figure S7). Specifically, the pores with sizes between 0.01 and 1 μm, which are initially present in PSA, are absent in PPC10 and PPC20, while only pores larger than 100 μm remain for PPC30. On the contrary, the micro/mesopore-size distribution obtained by the BJH method shows that the incorporation of PPy into PSA leads to a reduction of the amount of large mesopores (10–300 nm, in agreement with the MIP study) coupled with an increase in the amount of small mesopores (3–10 nm) independent of the amount of pyrrole used for the PPy layer formation (Figure S8).

The aforementioned observations suggest that the formation of PPy within the PSA framework starts to occur in the mesopores, and the growing PPy chains gradually form their own network. The partial closure of large mesopores to form smaller pores explains the reduction of the micro/mesopore volume (*V*_BET_) and of the macropores surface area (*S*_MIP_) as well as the increase of the surface area of the micro/meso-pores (*S*_BET_) of PPC10 compared to that of PSA (Table 1). The pore-closure effect due to the PPy formation is further confirmed by the fact that a higher proportion of pore volumes are detected at higher intrusion-pressures during the MIP analysis of the PPC samples (Figure S9). In the presence of more pyrrole, the growing PPy chains form their own network, to the extent of completely covering the PSA framework, thus changing the morphology of the resultant PPC samples. The hypothesis regarding the formation of new PPy networks is supported by (i) the SEM images that show an increasing deviation from the honeycomb structure of the PSA to the lamellarized structure when increasing concentration of pyrrole is used to prepare the PPC (Figure 1) as well as (ii) the EDS analyses that indicate the complete coverage of PSA surfaces by PPy (Figures S4–S6). The formation of PPy within the framework of PSA creates a loose PPy network with new macropores of larger sizes. This results in the increase of the porosity and macropore volume (*V*_MIP_) of PPC10 compared to PSA, while the surface area of macropores (*S*_MIP_) is lowered (Table 1). As the concentration of pyrrole increases, the PPy network becomes tighter due to stacking of the PPy granules to form consolidated films that result in a reduction of the effective porosity and *V*_MIP_ in PPC20 and PPC30 (Table 1).

**Table 1:** Pore Structure Parameters of the Cryogels^a^

sample	skeletal density^b^(g cm^–3^)	bulk density(g cm^–3^)	effective porosity (%)	MIP pore surface area,*S*_MIP_(m^2^ g^–1^)	BET pore surface area,*S*_BET_(m^2^ g^–1^)	MIP pore volume,*V*_MIP_(cm^3^ g^–1^)	BET pore volume,*V*_BET_(cm^3^ g^–1^)
PSA	1.67	0.345	79.30	23.974	5.7	2.21	0.352
PPC10	1.45	0.142	90.21	0.6680	10.6	4.94	0.051
PPC20	1.39	0.272	80.48	0.725	17.3	3.05	0.081
PPC30	1.29	0.637	50.81	0.008	17.5	2.21	0.085

a Note: Unless otherwise mentioned, the results were based on mercury porosimetry analyses.

b Results obtained using pycnometry

To understand the possible factors leading to such a significant morphological difference between the PPC samples, we measured both the gravimetric and volumetric swelling degrees (*Q*_w_ and *Q*_v_) of PSA cryogels in water–pyrrole solutions of various concentrations (Figure S10). It was found that the *Q*_w_ decreases monotonically with an increasing pyrrole concentration, while the *Q*_v_ plateaus once the pyrrole concentration reaches 20%. The reduced swelling degrees are due to the reduced osmotic pressure gradient between the PSA cryogel and the pyrrole solution when the latter concentration (and therefore its osmotic pressure) is increased. Note that, while the increase in *Q*_w_ represents the general uptake of the water into the PSA network as well as by the pores, the increase in *Q*_v,_ on the contrary, is mainly associated with the swelling of the polymer network. So, the reduction of the *Q*_v_ at higher pyrrole concentrations indicates a restricted uptake of water as well as of pyrrole molecules into the PSA network, with most of the pyrrole molecules remaining in the pores. Apart from the reduced osmotic pressure gradient, which is acting as the driving force for swelling, the restricted uptake of water into the PSA network when immersed in solutions of higher pyrrole concentrations might be also attributed to the possible infiltration of pyrrole molecules, which have a lower water-affinity, into the PSA cryogel, reducing its hydratability. As such, the plateau of the *Q*_v_ at a pyrrole concentration higher than 20% may be attributed to the equilibration between the swelling pressure of the pyrrole-infiltrated PSA cryogel network with the osmotic pressure of the precursor pyrrole solution. In contrast, for the case of PSA immersed in a solution of lower pyrrole concentration (10%), a greater portion of the pyrrole solution diffuses into the PSA network, thereby inducing greater volumetric swelling, while only a small portion of pyrrole remains in the pores. It is likely that such a difference in the spatial distribution of pyrrole, particularly at the 10 and 20% junctures, results in a shift from a honeycomb to a multilayered structured PPy layer.

Another plausible factor affecting the PPC morphology is the possible variation of the APS/pyrrole concentration ratio in PSA (after the dipping) when an increasing concentration of pyrrole solution (before the dipping) is used. As mentioned before, for the fabrication of PPCs, the PSA cryogels are first loaded with pyrrole through the dipping process. The pyrrole-treated PSA cryogels are then transferred into an APS solution in order to induce the polymerization of pyrrole into PPy. Although we have deliberately kept constant the initial concentration ratio of APS/pyrrole in the two solutions used for the PSA dipping, the APS/pyrrole ratio in the PSA may not necessarily be constant due to a reduced uptake of pyrrole despite having its concentration increased. The suppressed *Q*_v_ indicates that an increase in the pyrrole concentration may not result in a corresponding increase in the amount of pyrrole taken up into the PSA network. This inference is reasonable when we assume that the uptake of water into PSA network (which would be reflected by a corresponding increase in its *Q*_v_) occurs concurrently with the uptake of pyrrole into the PSA network. However, due to the similarity of the *Q*_v_ values (i.e., 7–12 cm^3^ cm^–3^), the amount of pyrrole taken up into the PSA network may be rather similar even when the precursor pyrrole concentration increases from 10 to 30%. This may have resulted in an inadvertent increase of APS/pyrrole ratios during the polymerization step. The higher APS concentration may then result in a faster polymerization rate where the polymerization of free pyrrole (in the pores of PSA) is favored compared to bound pyrrole (in the PSA network).^52^ This forms the multilayered structures observed in PPC20 and PPC30. Apart from the influence on the polymerization rate and the preferred monomers (i.e., those in the pores or those entrapped in the network) to be oxidized, the effect of the higher APS/pyrrole ratio on the morphology of the resultant PPC can be attributed to the preferred orientational arrangement of PPy growth during polymerization. For example, it was proposed that, when the ratio of APS/aniline is high, the parallel alignment of the polyaniline growing chain is dominant, which leads to the formation of compact and layered structures, while at a low APS/aniline ratio, the parallel alignment of the polyaniline growing chain was inhibited, which led to the formation of spherical PANI nanoparticles.^53^ Similar to our case, they have also observed that, as the ratio of APS/aniline increases, the morphology of the as-synthesized PANI evolves from small PANI nanoparticle clusters to densely packed PANI nanoparticles of a larger diameter to compact PANI layers.^53^

### 3.2 Chemical and Thermal Properties

**Figure 2:**
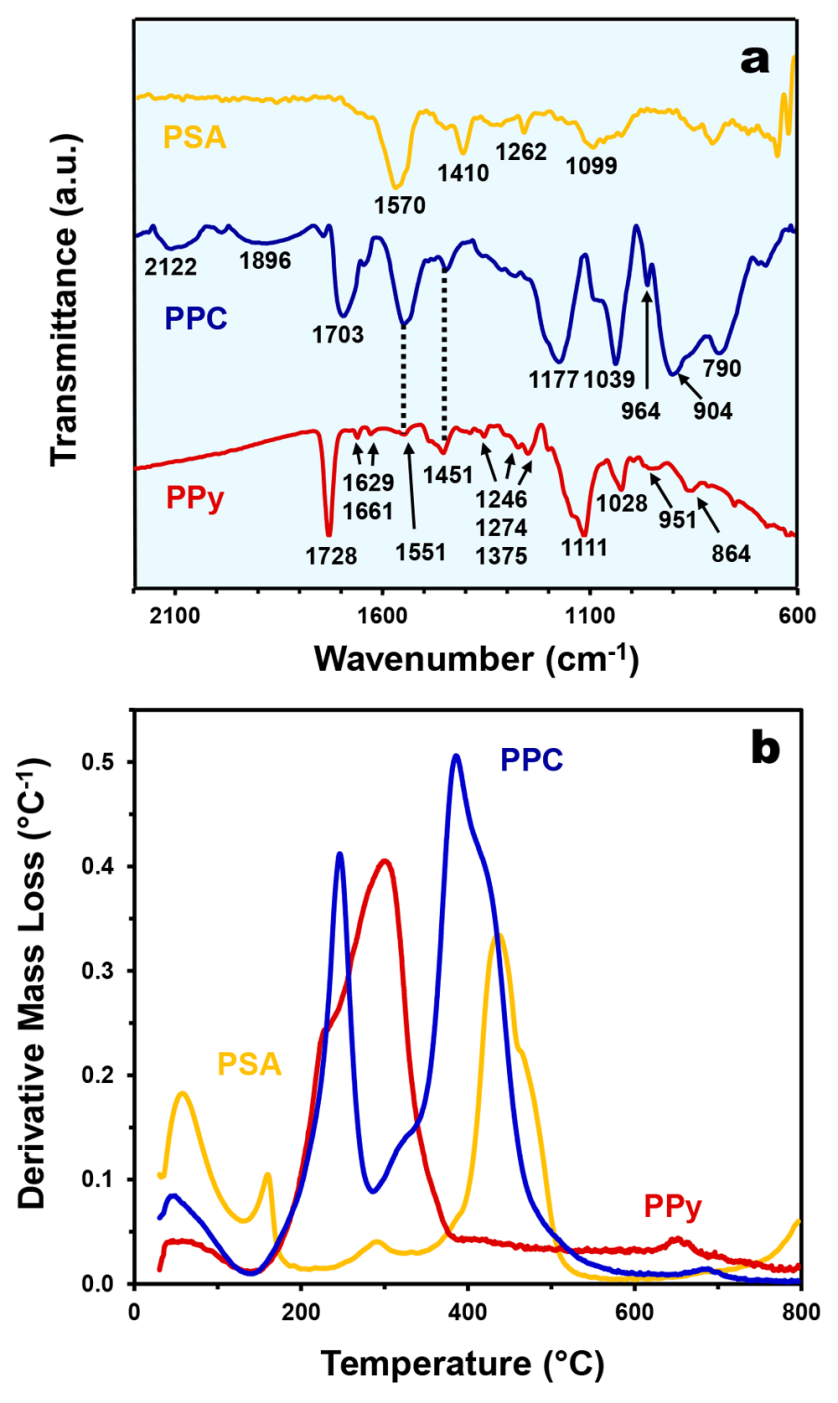
(a) Narrow-range FTIR spectra of the samples with annotated peaks. (b) Derivative thermogram of the samples. Note that PPC is represented by PPC10.

FTIR analysis was conducted to investigate the chemical interaction(s) between PSA and PPy in the PPC samples (PPC10 results are shown in Figure 2a; results for all samples are shown in Figure S11). For PSA samples, two strong absorption peaks were observed around 1570 and 1410 cm^–1^, which are assigned to the C=O stretching vibration of the carboxylate group (—COO^–^). The peaks at 1262 and 1099 cm^–1^ correspond to the C—O stretching vibration. The absorption bands at 2918 cm^–1^ can be assigned to —CH_2_— stretching, while the broad band at 3353 cm^–1^ may be attributed to the O—H stretching vibration of water present in the PSA.^4^ As for pure PPy, the FTIR spectrum also shows the presence of all its characteristics absorption peaks: 864 cm^–1^ (=C—H out-of-plane bending), 951 cm^–1^ (=C—H in-plane vibration), 1028 cm^–1^ (C—H deformation and N—H deformation vibrations), 1111 cm^–1^ (C—N stretching), 1274 and 1246 cm^–1^ (C—N stretching band and =C—H in plane bending) and 1375 cm^–1^ (C—N plane deformation), 1451 cm^–1^ (C—N pyrrole ring vibration), 1551 cm^–1^ (C=C, C—C ring stretching), 1661 and 1629 cm^–1^ (C=N stretching), 2870 cm^–1^ (aromatic C—H stretching vibrations), and 3308 cm^–1^ (aromatic N—H stretching vibration in pyrrole). Note that the distinct peak around 1728 cm^–1^ corresponds to the presence of a carbonyl group (C=O) formed by the nucleophilic attack of water during the PPy synthesis.^54,55^ Bands observed for PSA and PPy in the present study are consistent with those available in the scientific literature.^56−58^

In the FTIR spectrum for all PPC samples, most spectral features displayed by PSA are not apparent except from the methylene C—H asymmetric and symmetric stretching (arising from the aliphatic PSA backbone) at 2920 and 2852 cm^–1^, respectively (Figure S11). On the contrary, comparing the FTIR spectrum of PPC10 to that of PPy, there is a new peak at 1703 cm^–1^ that may be a red-shifted peak for the C=O vibration observed at 1728 cm^–1^ at the pure PPy spectrum. Furthermore, there is an intense peak at 1551 cm^–1^ that may be attributed to (i) a red-shifted C=O stretching of carboxylate (observed at 1570 cm^–1^ for PSA sample) due to its interaction with PPy in PPC10 and/or (ii) enhanced fundamental vibration of the pyrrole rings at (1551 cm^–1^). In addition, the appearance of new bands at 790, 1896, and 2122 cm^–1^, which are the aromatic group vibration frequencies, indicate that the presence of PSA as a dopant may have also enhanced charge delocalization into the pyrrole rings.^59^ Note that the bands at 1896 and 2122 cm^–1^ are the overtones of the out-of-plane C—H bending as evident by the bands at 790, 904, and 964 cm^–1^. Another difference observed at the PPC10 sample compared to the spectrum of pure PPy is the blue shift of the peaks attributed to C—N stretching from 1111 to 1177 cm^–1^ and from 1028 to 1039 cm^–1^. This, in addition to the disappearance of the N—H stretching band of the PPy, the disappearance of the peaks for the carboxylate group vibrations of PSA and the, possible, red-shifted C=O stretching of carboxylate of PSA in the PPC10 spectrum indicate that the N—H groups in pyrrole rings are hydrogen bonded to the carboxylate groups of PSA.

The thermal properties of the samples were studied through thermogravimetric analysis. As shown in Figure 2b, besides the weight loss due to sorbed moisture, PSA has three major decomposition peaks with *T*_max_ values at 160, 290, and 440 °C with weight losses of 6.54, 4.08, and 22.4%, respectively (Figure S12). The first decomposition step can be attributed to the release of constitutional water. As the temperature progressively rises up to 340 °C, the weight loss can be attributed to the intermolecular dehydration reaction that forms anhydrides. Finally, within a temperature range of 340–550 °C occurs the major fragmentation of the PSA backbone including the decomposition of side chains.^60,61^ As for PPy, its onset decomposition temperature starts at 140 °C and ends at 390 °C with a weight loss percentage of 48.9% that is typical of PPy formed using APS as oxidant.^62^ Apart from the weight loss due to the release of constitutional water, the PPC samples show all the major decomposition peaks of the individual PSA and PPy components coupled with a shift to lower *T*_max_ values of 245 and 385 °C with weight loss percentages of 23.5 and 55.9%, respectively. In addition, it is noted that the decompositions occurring at *T*_max_ of 245 and 385 °C take place with an enhanced rate, as evidenced by the sharper and taller peaks, indicating that the thermal stabilities of both PSA and PPy are reduced when incorporated into PPC. In fact, it has been observed by others that the incorporation of PPy has a negative impact to the thermal stability of the hydrogel substrate.^63^ Nonetheless, the photoconverted heat in solar evaporators rarely exceeds 100 °C, thus the PPC samples are mostly stable within the working temperature range for solar evaporation. Another important point from the differential thermogravimetric analysis is the fact that there is no formation of new decomposition peaks, indicating that PSA and PPy did not chemically react to form a new compound in PPC. This corroborates with the findings from FTIR that suggests that PSA and PPy interact with each other mainly *via* hydrogen bonding.

### 3.3 Water-Absorption Properties

**Figure 3:**
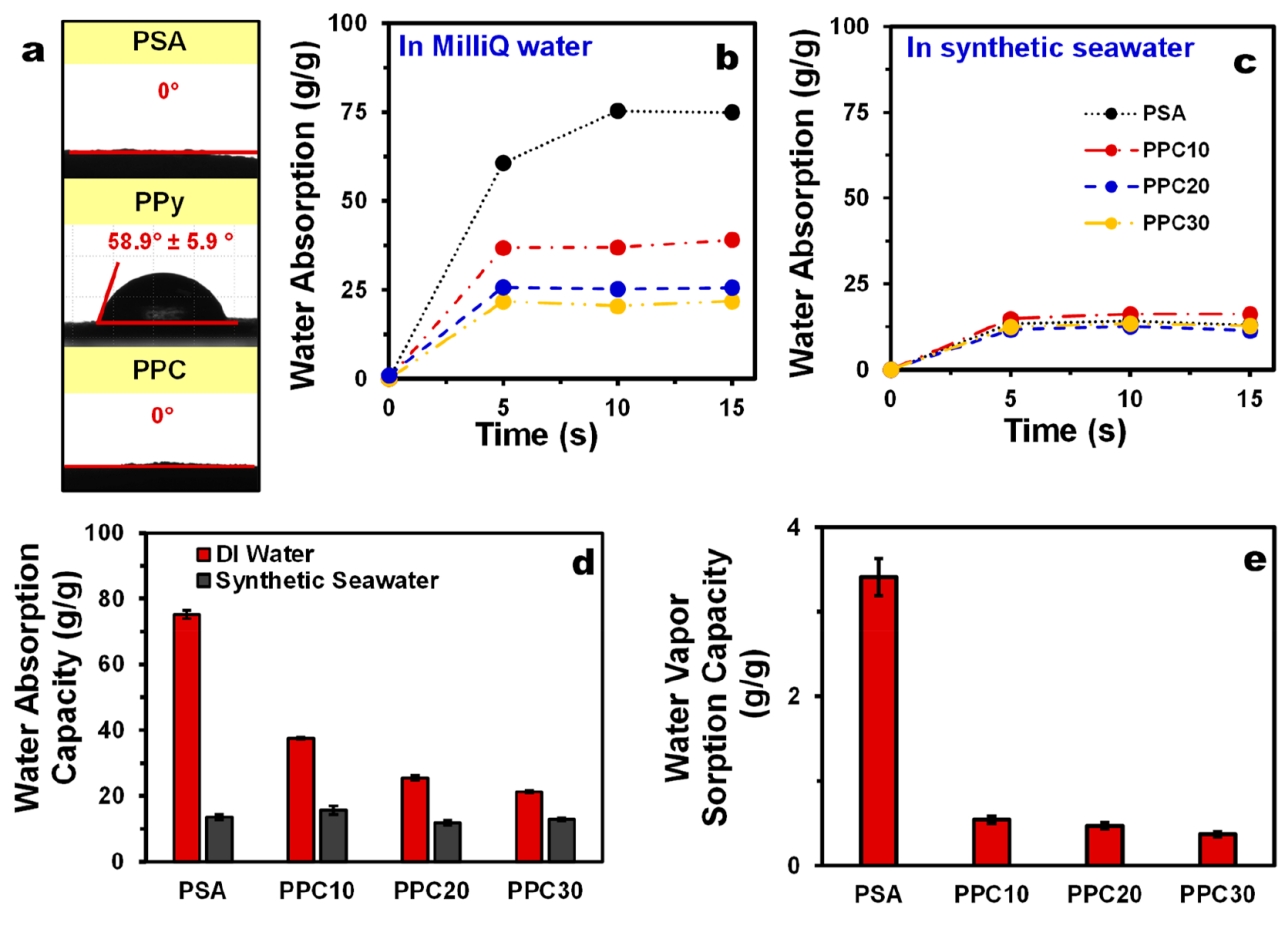
(a) Static water contact angles of the samples. Dynamic water-absorption profiles for the samples in (b) Milli-Q water and (c) synthetic seawater (3.5 wt %). (d) Maximum water-absorption capacities of the samples in Milli-Q water and synthetic seawater. (e) Water vapor sorption capacities of the samples.

Generally, for the solar evaporator systems, the continuity and the rate of the solar evaporation process is contingent upon the efficient bottom-to-top water transport from the bulk to their evaporative surface to replenish the evaporated water. As such, to evaluate the applicability of the PPCs as solar evaporators, their water-wetting and -absorption properties were studied. As shown in Figure 3a, although the wettability of a PPy film is significantly lower than that of the PSA cryogel as indicated by the higher static water contact angle (59° versus 0°), PPC samples still retain the superwetting property of the PSA cryogel regardless of the presence of the PPy component. Importantly, for all PPC samples, the water droplet is absorbed slightly faster compared to the pure PSA cryogel (0.08 versus 0.16 s) (video stills shown in Figure S13 in the Supporting Information). The slower water-droplet absorption by the PSA cryogel may be due its lower surface roughness and its more compact network, as suggested by its higher skeletal density (Table 1), which may prohibit rapid water uptake. This corroborates with the dynamic water-absorption profile of PSA cryogel, which took 10 s to reach its equilibrium swollen state, while that of the PPC samples can be achieved within 5 s (Figure 3b). Although the equilibrium swollen state is reached faster for all PPC samples, the increasing pyrrole concentration in the PPC samples lowers the water-absorption capacity compared to that of pure PSA cryogels due to the lower water affinity of PPy (Figure 3d).

The water-uptake properties of the samples were also investigated using synthetic seawater as they are intended to be applied for solar desalination (Figure 3c). When the samples were allowed to swell in synthetic seawater, they still retain their rapid swelling abilities, but their water-absorption capacities are significantly diminished due to the high ionic strength of the solution, although no effect was observed for the kinetics. The suppression of the water-absorption capacity is most severe in the PSA cryogel because of its high charge-density due to the numerous carboxylate groups in its network. On the contary, it is less severe in the PPC samples because, as suggested by the FTIR results, the carboxylate groups from PSA are interacting with the N–H groups in pyrrole rings, thereby reducing its charge density. In fact, all the gels show similar water-absorption capacities, in the range 12–16 g g^–1^, in synthetic seawater (Figure 3c).

In terms of their ability to absorb moisture, it was found that the water-vapor-absorption capacity is significantly reduced compared to that of PSA cryogels. While the PSA cryogels show 340% increase in weight due to moisture absorption, the PPC samples show only 37–54% increase (Figure 3e). This shows that the incorporation of PPy, which has a much lower water-affinity compared to PSA, has a strong impact on the equilibrium water-uptake properties of their resultant hybrid. The reduced water-absorption capacity of the PPC samples endows them with a self-floating ability for up to 72 h in contact with water (Figure S14). However, this is not the case for the PSA cryogels, which could not float due to the absorption of a large amount of water. In addition, the moderate interaction between PPC and water may actually facilitate the water evaporation from their structure as it is an energetically less demanding process.^64^ Furthermore, it has been recently found that the downward conductive heat loss is minimized when the water-absorption rate is not too much higher than the evaporation rate of the materials.^29^

### 3.4 Photothermal Properties

The efficient absorption of solar radiation and its conversion to heat is a critical step to drive water evaporation. As only the photons absorbed can potentially contribute to the photothermal transduction, it is important that solar evaporators display high photoabsorption across the broad solar spectrum. Thus, the light absorption of PPC disks (with thickness ca. 2 mm) was measured from 200 to 2500 nm. The PPC samples show a relatively good light absorption within the studied wavelength range. Specifically, PPC10 absorbed 88%, while both PPC20 and PPC30 absorbed about 78–80% of the light in the whole wavelength range studied (Figure S15a). As all the samples exhibited negligible transmittance, the lower light-absorption efficiencies of PPC20 and PPC30 are mainly due to their higher diffused reflectance (Figure S15b). Note that, while the doped PPy has a good intrinsic light absorption owing to the enhanced absorption in the NIR region (partly due to the presence of bipolarons),^65^ the light-absorption property of PPCs is also influenced by the light-trapping ability that depends on their morphology.^27^ As such, the higher reflectance observed for PPC20 and PPC30 can be attributed to the lower roughness of their surfaces, which are composed of smoother and flatter planar sheets compared to PPC10, that resulted in a substantial loss of reflected light. In contrast, PPC10 has a honeycomb structure with open pores coupled with a highly rough pore surface due to the presence of PPy particles. This type of 3D structure can effectively retain and redistribute the incident light *via* trapping of transmitted light, causing multiple internal light scattering in confined spaces thus resulting in the low reflectance loss.^66^

**Figure 4:**
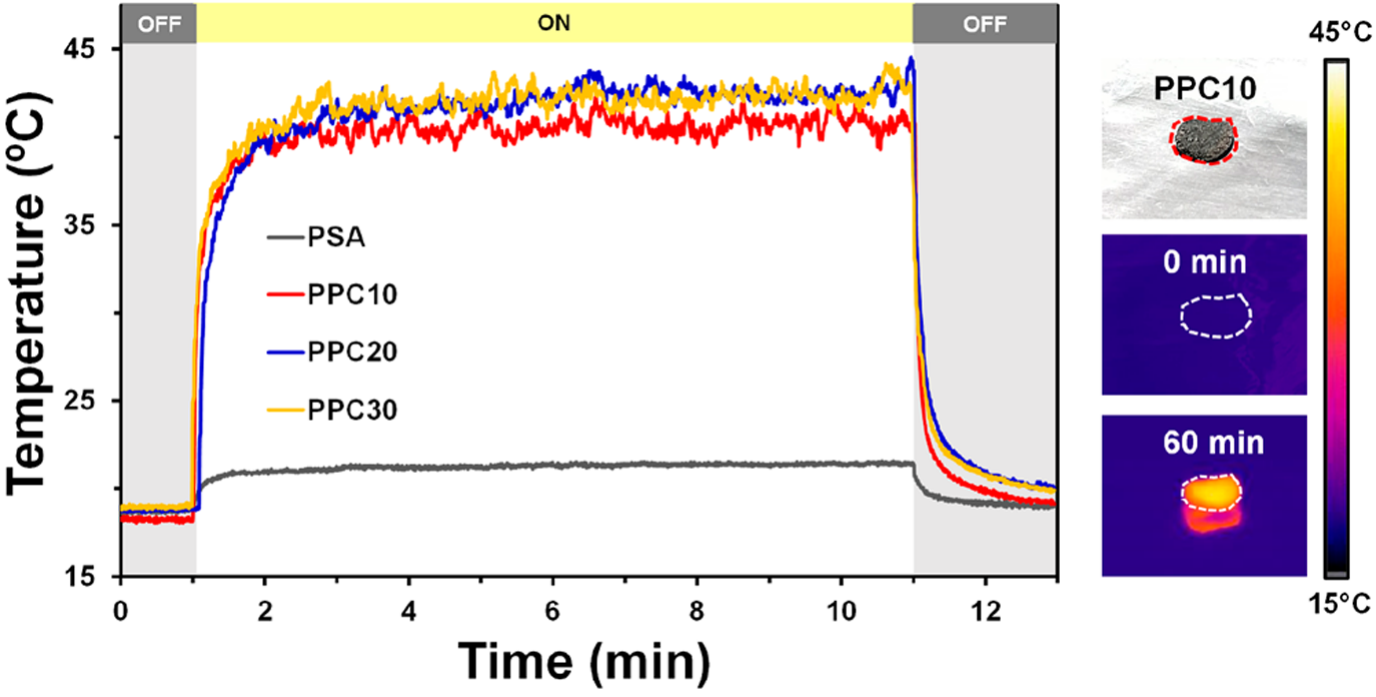
IR thermal imaging of the dry PPC samples showing their photothermal conversion ability when exposed to 1 Sun irradiation.

Due to their strong light-absorption property, the surface temperature of dry PPC samples rapidly increases by 23–26 °C within 3 min of 1 Sun irradiation indicating their efficient photothermal transduction efficiency (Figure 4). In contrast, the surface temperature of a dry PSA cryogel only increased by 2.5 °C when irradiated under 1 Sun. This shows that the incorporation of PPy into a PSA cryogel significantly enhanced its photothermal transduction efficiency owing to the fact that the PPy can convert the absorbed incident photons to heat *via* nonradiative relaxation and molecular vibrations in its polymers chains.^26^ Furthermore, the low thermal-conductivity of the developed materials (0.033–0.038 W m^–1^ K^–1^) contributes to the minimization of the heat loss to the environment (Figure S16).

### 3.5 Solar Evaporation and Desalination Performance

**Figure 5:**
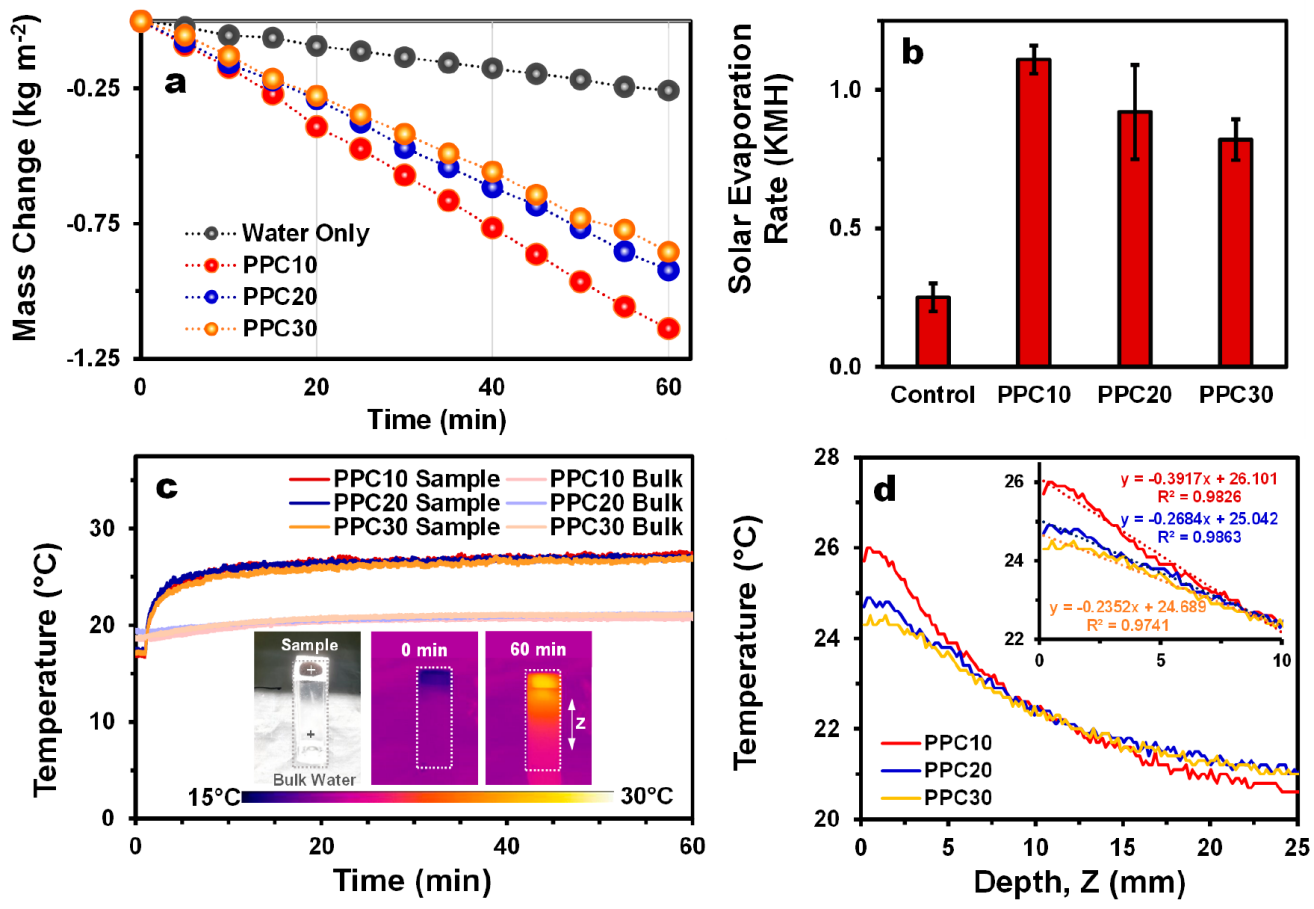
(a) Cumulative mass loss of water and (b) solar evaporation rates of water only and of water through the PPC samples due to evaporation under 1 Sun irradiation. (c) Temperature profiles of the sample surface versus bulk water as determined from IR thermal imaging; the insets show the digital photograph of the sample and the corresponding thermal images before and after 1 Sun irradiation. (d) Temperature gradient along the *z*-axis of the PPC samples, i.e., from the sample midpoint to the bulk water, showing the difference in heat localization efficiency.

Due to their low density and lower water-content, the PPCs can self-float on the top surface of water, thus enabling the localization of phototransduced heat on their interface for efficient evaporation. The solar evaporation rates (SERs) of the PPC samples under 1 Sun were determined by measuring the cumulative mass loss of water through the PPC samples. As a reference control, the SER of water under 1 Sun was also determined. As shown in Figure 5a, the mass loss of Milli-Q water through PPC samples occurs significantly faster compared to that in the absence of the samples. Specifically, the SER for the control was determined to be 0.25 KMH (kg m^–2^ h^–1^), while the SERs of PPC10, PPC20, and PPC30 were 1.11, 0.92, and 0.82 KMH, which are 4.44, 3.68, and 3.28 times higher than that of the control, respectively (Figure 5b). Using eq 3, the solar-to-vapor conversion efficiencies of PPC10, PPC20, and PPC30 are, therefore, 76.3, 63.0, and 56.1%, respectively. Although the PPC samples show a similar photothermal conversion efficiency as evidenced by their dynamic temperature profile upon irradiation under 1 Sun (Figure 4), PPC10 displays a higher SER compared to other PPC samples.

To understand the factors engendering the differences in the SERs, we studied the heat localization effect in the PPC samples by recording the dynamic heat distribution of the PPC samples under the same conditions for the SER measurement *via* IR thermal imaging. As shown in Figure 5c, before solar irradiation, the PPC samples have a lower temperature than the bulk water due to evaporation of water in the dark. Once they are irradiated under 1 Sun, their surface temperature rapidly increases, and within 10 min, the PPC samples reach their plateau temperature, which is 9.4–10.5 °C higher than their initial temperature. In contrast, the bulk water for all samples shows little increase in temperature (1.8–2.3 °C) even after 60 min of solar irradiation. As shown in Figure 5d, a closer examination of the temperature gradient along the *Z*-axis from the midpoint of the PPC sample to the bulk water (as indicated in the inset of Figure 5c) reveals that the temperature gradient in PPC10 is the highest, indicating its superior heat localization effect. The enhanced heat localization effect in PPC10 may be ascribed to its well-interconnected polymer network with moderate-sized macropores that facilitates the compartmentalization of heat compared to PPC20 and PPC30, which possess lamellar structures with large gaps.

We have also analyzed the extent of heat losses in the three PPC samples. It was found that they have similar heat losses that varied between 12.1 and 12.9% due to conduction (2.3–3.3%), radiation (5.3–5.5%), and convection (3.8–4.4%) (details in the Supporting Information). This means that 87.8–87.1% of the absorbed light is used for vapor generation. So, while the heat losses are similar for all samples, their differences in SERs may be, in part, attributed to their difference in light-harvesting ability. In fact, when the photoabsorption efficiencies are taken into account into the heat loss analyses, the resultant energy efficiencies are generally in close agreement with that of the calculated solar-to-vapor conversion efficiencies (details in the Supporting Information). One of the reasons why PPC10 shows a higher SER is due to the superior light-harvesting properties arising from its pores with a higher surface-roughness compared to the smooth lamellar structures in PPC20 and PPC30 that show high light-reflectance (Figure S15b). However, it is noted that, for PPC20 and PPC30, the solar-to-vapor conversion efficiencies are slightly lower than the energy efficiency determined from the heat loss analysis. This indicates that the evaporation rate, which comprises both the generation and removal of vapor from their surfaces, is lower than their heat utilization efficiency. This underscores the importance of having open and well-interconnected pores to facilitate vapor escape. The predominance of closed pores in PPC20 and PPC30 may have hindered the efficient removal of the vapor generated, rendering their SER inferior to that of PPC10. In summary, the superior SER of PPC10 compared to those of PPC20 and PPC30 can be attributed to its (i) higher photoabsorption efficiency, (ii) higher heat localization effect enabling the efficient confinement of the photoconverted heat within its structure, (iii) open porous structures that facilitate vapor removal, (iv) rough pore surfaces and therefore higher surface area for light absorption and water evaporation, and (v) higher water-absorption capacity to ensure efficient water replenishment to the evaporative sites, ensuring continuous evaporation while maintaining a high heat-transfer rate.^67^

**Figure 6:**
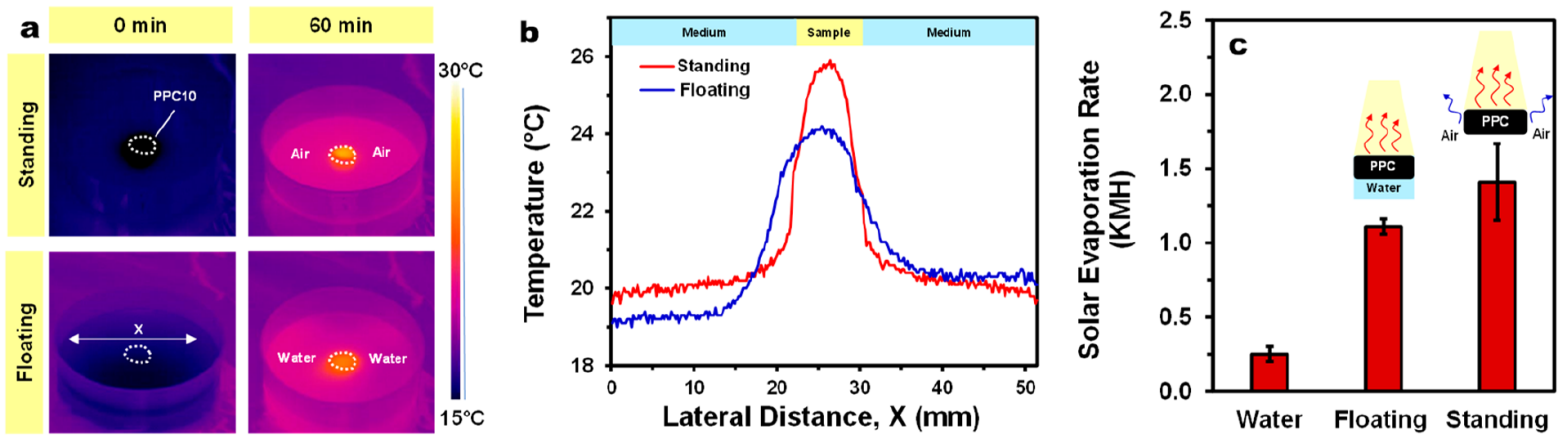
(a) Thermal images of PPC10 comparing their temperature distribution when irradiated with 1 Sun under floating or standing modes. (b) Temperature gradient between PPC10 and the environment (i.e., air or water) across the lateral axis, *x*. (c) Solar evaporation rates of PPC10 in different testing modes.

Due to the ability of PPC10 to retain the absorbed water, we also measured their SER in the standing mode, without constant contact with water (as in the case for the floating mode) but only with the water that was absorbed into the sample prior to 1 Sun irradiation. To study the difference in the temperature distribution between the sample and the environment (i.e., air or water) for the standing mode compared to the floating mode, the lateral *x*-axis temperature gradient for the two modes was recorded *via* IR thermal imaging (Figure 6a,b). As shown in Figure 6b, the standing mode resulted in a more uniform temperature distribution within the sample, and most of the photoconverted heat is mainly confined within the sample with minimal heat loss to ambient air. On the contrary, the floating mode shows a lower surface temperature in tandem with a greater lateral heat loss to the surrounding bulk water. Furthermore, the temperatures of water on both sides of the floating sample are different. This indicates that the standing mode is better than the floating mode in terms of heat management, particularly for systems where conductive heat loss to bulk water is severe.

The apparent SER determined for the standing mode is 1.41 KMH with a solar-to-vapor conversion efficiency of 96.9% (Figure 6c). Note that we have used the term “apparent” here because the SER is conventionally calculated on the basis of the illuminated area, which would suffice for the floating mode as the lateral surfaces are submerged in water, but for the standing mode, the lateral surface area due to the exposed side walls is not included in the calculation (see inset in Figure 6c). The apparent SER, without accounting for the lateral surface area, of the standing mode is 27% higher than that of the floating mode. However, when the lateral surface area is included in the calculation, the SER of the standing mode becomes about 44% lower than that of the floating mode. This indicates that the higher apparent SER of the standing mode arising from the lateral surfaces is not of pure photothermal origin. Note that, even though some regions cannot be directly illuminated, thermal energy can still be transferred to these regions when the top surface is exposed to solar irradiation.^68^ Although the higher heat localization effect observed in the standing mode may have also led to the enhanced apparent SER, the effect is most likely marginal compared to the contribution from the evaporation from the side walls as the conductive heat loss in the floating system is already quite low (i.e., 3.1%; details in the Supporting Infrmation). Thus, it is expected that the apparent SER of PPC10 can be further enhanced by increasing its thickness to increase the lateral surface for evaporation.^30^ In fact, it is likely that, when the height is substantially increased while maintaining a good water-uptake, the temperature of the bottom part of the cylinder may be reduced to be even lower than the ambient environment.^69−71^ In such cases, instead of losing thermal energy to its environment, energy can be gained to facilitate “natural” evaporation from the side walls. This strategy will not only improve the apparent SER but also it enables evaporation to occur even on low solar-flux days.

**Figure 7:**
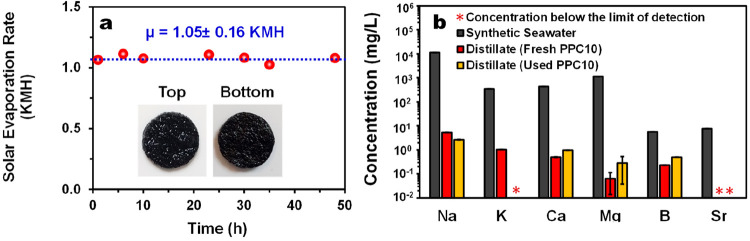
(a) Long-term solar evaporation of PPC10 in synthetic seawater under 1 Sun irradiation tested in the floating mode; the insets are the photographs of PPC10 after the 48-h test showing the absence of salt accumulation on both sides of the sample (top surface is exposed to solar irradiation). (b) Concentration of the major elements, as determined using inductively coupled plasma-optical emission spectrometry (ICP-OES), in synthetic seawater (3.5 wt %) and in the distillate collected using fresh PPC10 or PPC10 after being used for solar evaporation under 1 Sun in synthetic seawater for 24 h.

Apart from the efficient short-term performance, an effective solar evaporator should also (i) present a stable operation in prolonged irradiation conditions, (ii) be salt-resistant, and (iii) present high desalination efficiency. The long-term solar evaporation performance of synthetic seawater (3.5 wt %) was examined using our best performing sample, PPC10. As shown in Figure 7a, PPC10 could maintain an average SER (μ) of 1.05 ± 0.16. The performance is not only similar to the short-term (1 h) SER of PPC10 in Milli-Q water (in the floating mode) but also rather stable. As shown in the inset of Figure 7a, no visible salt accumulation on the surface of PPC10 could be observed even at the end of the 48-h test. This is further proven by the SEM imaging, where it is shown that, although the pores of the used PPC10 appear to be smaller, no large salt crystals on its surface are observed (Figure S17a,b). The pristine morphology of the PPC10 is completely restored after being left to stand in Milli-Q water for 1 h (Figure S17c).

Further examination of the anticlogging property of PPC10 was conducted *via* EDS analyses. Figures S18 and S19 in the Supporting Information show the EDS spectra of the surface and cross section, respectively, of dried samples of PPC10 before and after the 48-h solar evaporation in synthetic seawater and after washing the used sample in water. As revealed, in addition to the main pristine components of PPC10, the Na, Mg, Ca, Cl, and K elements are present on the surface and cross-section of the used PPC10 sample. However, a significant reduction in these signals is observed in the EDS spectra of the used PPC10 after washing. These evidence suggest that the anticlogging property of PPC10 may be due to the reduced local salt concentration at the evaporating surface, attributable to the combined action of (i) the high water-content present in its skeleton that could prevent the supersaturation of salts^36^ and (ii) its macroporous structure that may promote the dilution of salt solutions at the evaporating interface *via* back diffusion to the bulk solution.^72−74^ It should be mentioned that, since PPy coats thoroughly the PSA cryogels, the high charge-density of the PSA that was expected to contribute to the salt-rejecting properties in the PPC through the Donnan exclusion effect^42−44^ may be suppressed in our case.

The reusability of PPC for solar desalination was also evaluated by collecting the condensed vapor generated by the solar evaporation of a synthetic seawater (3.5 wt %) through fresh PPC10 and PPC10 samples previously used for 24 h of solar evaporation in synthetic seawater. The concentrations of the major elements (Na, K, Ca, Mg, B, and Sr) were analyzed using ICP-OES. Compared to the synthetic seawater, the concentrations of all the aforementioned elements in the distillate collected using fresh and used PPC10 samples are significantly reduced (Figure 7b). In particular, the concentration of Na is reduced by at least 4 orders of magnitude while other elements are reduced to a subppm level or nondetectable. Note that the higher concentration of Na in the distillate compared to Ca and Mg may be attributed to the significantly lower hydration energy of Na^+^ (−365 kJ mol^–1^) compared to those of Ca^2+^ (−1505 kJ mol^–1^) and Mg^2+^ (−1830 kJ mol^–1^).^75^ In fact, Ca^2+^ and Mg^2+^ are highly hydrated as they are surrounded by 7.2 and 10 water molecules in their respective hydration shells compared to that of Na^+^, which is only surrounded by 3.5 water molecules.^75^ As such, the evaporation of the large Ca^2+^ and Mg^2+^ ion–water clusters may not be energetically favorable as the interaction between the nonvolatile salt ions and the more-volatile water molecules is stronger compared to the interaction between the water molecules.^76,77^ This could be the reason that the concentrations of Ca and Mg are significantly lower than that of Na in the distillate. On the contrary, although the hydration energy of K^+^ (−295 kJ mol^–1^) is rather similar to that of Na^+^,^75^ the concentration of Na in the synthetic seawater is about 1.5 order of magnitude higher than that of K possibly due to the higher concentration of Na in the distillate. Other studies have also reported a similar trend where the concentration of Na is higher than those of Mg and K in the distillate.^78−80^ For the case of B, which typically exists in seawater in the form of H_3_BO_3_,^81^ it becomes volatile at relatively high temperatures.^81,82^ Therefore, it is plausible that elevated temperatures in the localized interfaces within the PPC10 framework may have induced the evaporation of a small amount of H_3_BO_3_ together with water vapor, thereby resulting in a slightly higher concentration of B compared to Ca, Mg, and K in the distillate. Nonetheless, the high quality of the resultant distillate even after repeated use of the PPC10 further confirms their reusability and potential long-term utilization.

## 4 Conclusions

Photothermal cryogels have been successfully fabricated *viain situ* oxidative polymerization of pyrrole using APS as the oxidant and PSA as both the substrate and the dopant. The concentration of the precursor pyrrole used was found to have a significant effect on the morphology of the resultant PSA/PPy cryogel (PPC). As the pyrrole concentration used for fabrication was increased, the morphology of PPy evolved from nanoparticles to lamellar sheets and finally into consolidated thin films. The solar evaporation of the PPC fabricated using the lowest pyrrole concentration (i.e., PPC10) displayed the best rate. When operated in the floating mode, the solar evaporation rate was 1.11 kg m^–2^ h^–1^, which is 4.44 times higher than that of the pure water, with a solar-to-vapor conversion efficiency of 76.3%, while in the standing mode, the apparent solar evaporation rate and solar-to-vapor conversion efficiency increased to 1.41 kg m^–2^ h^–1^ and 96.9%, respectively, mainly due to the contribution of nonphotothermal evaporation from the exposed lateral surfaces. The distillate obtained from condensed vapor generated *via* the solar evaporation of a synthetic seawater through PPC10 shows at least 99.99% reduction of Na, while all the other elements were reduced to a subppm level. We attribute the superior solar evaporation and desalination performance of PPC10 compared to other PPC samples to their (i) higher photoabsorption efficiency, (ii) higher heat localization effect enabling the efficient confinement of the photoconverted heat within its structure, (iii) open porous structures that facilitates vapor removal, (iv) rough pore surfaces that increases the surface area for light absorption and water evaporation, and (v) higher water-absorption capacity to ensure efficient water replenishment to the evaporative sites ensuring continuous evaporation while maintaining a high heat-transfer rate.

## Supporting Information Available

Discussion of heat loss analyses, figures of schematic of the condensation chamber, digital photographs of the cryogel samples, SEM images, SEM-EDS mapping, pore-size distribution, mercury intrusion curves, gravimetric and volumetric swelling degrees, wide-scan FITR spectra, thermogram showing mass losses, video stills, UV–vis–NIR absorption and reflectance spectra, thermal conductivity, and EDS spectra, and table of composition of the synthetic seawater (PDF)
