# Supplementary material for: 3D Photothermal Cryogels for Solar-Driven Desalination
Source: ACS Appl Mater Interfaces. 2021 Jun 22;13(26):30542–55. doi: 10.1021/acsami.1c05087 (PMC8289246; doi:10.1021/acsami.1c05087)
Supplement: Supplementary file 1 — am1c05087_si_001.pdf [file am1c05087_si_001.pdf]

Supporting Information

3D Photothermal Cryogels for Solar-Driven Desalination

Siew-Leng Loo,*[a] Lía Vásquez,[ab] Muhammad Zahid[a], Federica Costantino[ac], Athanassia Athanassiou[a] and Despina Fragouli*[a]

[a]Smart Materials, Istituto Italiano di Tecnologia, Via Morego 30, 16163 Genova, Italy
[b]Dipartimento di Chimica e Chimica Industriale (DCCI) Università degli Studi di Genova, Via Dodecaneso 31, 16146 Genova, Italy
[c]Interdisciplinary Laboratories for Advanced Materials Physics (i-LAMP) and Dipartimento di Matematica e Fisica, Università Cattolica del Sacro Cuore, Via Musei 41, 25121 Brescia, Italy

*Corresponding authors: Email: Despina.Fragouli@iit.it; Siew.Loo@iit.it

S1. Supplementary Note

S1.1 Heat loss analyses

S2. Supplementary Figures and Table

S2.1 Schematic of the condensation chamber.
S2.2 Digital photographs of the cryogel samples.
S2.3 SEM showing the different morphological evolution from PSA to PPC30.
S2.4 SEM-EDS elemental mapping for the top surface of samples.
S2.5 SEM-EDS elemental mapping for the bottom surface of samples.
S2.6 SEM-EDS elemental mapping for the cross-sectional surface of samples.
S2.7 MIP pore-size distribution of the samples.
S2.8 BJH pore-size distribution of the samples.
S2.9 Mercury intrusion curves of the samples.
S2.10 Swelling degrees of PSA cryogels in pyrrole solutions of varying concentrations.
S2.11 Thermogram of the samples.
S2.12 FTIR spectra of all samples.
S2.13 Video stills from contact angle determinations of PSA and PSA/PPy.
S2.14 Digital photographs showing the floatability of PPC10.
S2.15 UV-vis-NIR absorption spectra of the PPC samples.
S2.16 Thermal conductivity of the samples.
S2.17 Anticlogging property of PPC10.
S2.18 EDS analyses of the outer surface of PPC10 for salt-resistance study.
S2.19 EDS analyses of the cross-sectional surface of PPC10 for salt-resistance study.
S2.20 Typical composition of the synthetic seawater

S1. Supplementary Note

S1.1 Heat loss analyses

PPC10

Conductive heat loss

Conductive heat flux (J_{cond}) from PPC10 (in the floating mode) to the bulk water can be determined based on the following equation:

$$J_{cond} = Cm\Delta T_{bulk} = 28.98\ \text{J} \qquad \text{Equation (S1)}$$

Where C is the specific heat capacity of water (4.2 kJ °C^{-1} kg^{-1}), m represents the weight of bulk water (3 g), and ΔT_{bulk} (20.8 – 18.5°C) represents the increased temperature of the bulk water after stable steam generation.

Energy input during solar evaporation, $E_{in} = I_{in} \times A \times t = 936$ J

Where A is the irradiated area of PPC10 (0.000064 m^2) and t is the time duration of the solar evaporation (3600 s).

Conductive heat loss = $J_{cond}/E_{in} \times 100\%$ = 28.98 J/936 J × 100% = **3.1%**

Radiative heat loss

Radiative heat transfer can be calculated using the Stefan-Boltzmann equation:

$$\Phi = \varepsilon A\sigma\ (T_{PPC10}^4 - T_{ambient}^4) \qquad \text{Equation (S2)}$$

Whereby Φ denotes the heat flux, ε is the emissivity, A is the area of the evaporation surface (0.000064 m^2), σ is the Stefan-Boltzmann constant (5.67x10^{-8} W m^{-2} K^{-4}), T_{PPC10} and $T_{ambient}$ are the temperatures of PPC10 (300.4 K) and ambience (291 K), respectively. Since PPC has a relatively high light absorption, it can be treated as a black-body in thermal equilibrium with an ε of 1.

Radiation flux, $J_{rad} = \Phi/A = \varepsilon\sigma\ (T_{PPC10}^4 - T_{ambient}^4)$ = 55.1 W m^{-2}

Radiative heat loss = $J_{rad}/I_{in} \times 100\%$ = 59.4 W m^{-2}/1000 W m^{-2} × 100% = **5.51%**

Convective heat loss

Convective heat transfer, P_{conv} can be calculated by the Newton's law of cooling:

$$P_{conv} = hA(T_{PPC10} - T_{ambient}) \qquad \text{Equation (S3)}$$

Where h is the convective heat transfer coefficient (5 W m^{-2} K^{-1}).

Convective flux, $J_{conv} = P_{conv}/A = h(T_{PPC10} - T_{ambient})$ = 37.6 W m^{-2}

Convective heat loss = $J_{conv}/I_{in} \times 100\%$ = 50 W m^{-2} /1000 W m^{-2} × 100% = **3.76%**

Therefore, the total heat loss of PPC10 (in the floating mode) is **12.37%** (3.1% + 5.51% + 3.76%) under 1-Sun illumination. This means that 87.6% of the absorbed light, which is ca. 88% of the power input, is used for vapor generation. As such, based on this analysis, 77.1% of the solar input was used to generate vapor. This value is in close agreement with the value of solar-to-vapor conversion efficiency determined based on the solar evaporate of PPC 10 which is found to be 76.3%. This indicates that the rate of vapor generation and removal from the surface of PPC10 matches its photothermal conversion efficiency. Further enhancement of the solar evaporation rate can be achieved via enhanced light harvesting ability.

PPC20

Conductive heat loss

Conductive heat flux (J_{cond}) from PPC20 (in the floating mode) to the bulk water can be determined based on Equation S1:

$$J_{cond} = Cm\Delta T_{bulk} = 21.42\ \text{J}$$

Where C is the specific heat capacity of water (4.2 kJ °C^{-1} kg^{-1}), m represents the weight of bulk water (3 g), and ΔT_{bulk} (21.2 – 19.4°C) represents the increased temperature of the bulk water after stable steam generation.

Energy input during solar evaporation, $E_{in} = I_{in} \times A \times t = 936$ J
Where A is the irradiated area of PPC20 (0.000064 m^2) and t is the time duration of the solar evaporation (3600 s).

Conductive heat loss = $J_{cond}/E_{in} \times 100\% = 21.42\ \text{J}/936\ \text{J} \times 100\%$ = **2.29%**

Radiative heat loss

Radiative heat transfer can be calculated using the Stefan-Boltzmann (Equation S2):

$$\Phi = \varepsilon A\sigma\ (T_{PPC20}^4 - T_{ambient}^4)$$

Whereby Φ denotes the heat flux, ε is the emissivity, A is the area of the evaporation surface (0.000064 m^2), σ is the Stefan-Boltzmann constant (5.67x10^{-8} W m^{-2} K^{-4}), T_{PPC20} and $T_{ambient}$ are the temperatures of PPC20 (300.1 K) and ambience (291 K), respectively. Since PPC has a relatively high light absorption, it can be treated as a black-body in thermal equilibrium with an ε of 1.

Radiation flux, $J_{rad} = \Phi/A = \varepsilon\sigma\ (T_{PPC20}^4 - T_{ambient}^4) = 53.3$ W m^{-2}
Radiative heat loss = $J_{rad}/I_{in} \times 100\%$ = 53.3 W m^{-2}/1000 W m^{-2} × 100% = **5.33%**

Convective heat loss

Convective heat transfer, P_{conv} can be calculated by the Newton's law of cooling (Equation S3):

$$P_{conv} = hA(T_{PPC20} - T_{ambient})$$

Where h is the convective heat transfer coefficient (5 W m^{-2} K^{-1}).

Convective flux, $J_{conv} = P_{conv}/A = h(T_{PPC20} - T_{ambient}) = 45.5$ W m^{-2}
Convective heat loss = $J_{conv}/I_{in} \times 100\%$ = 45.5 W m^{-2} /1000 W m^{-2} × 100% = **4.45%**

Therefore, the total heat loss of PPC20 (in the floating mode) is **12.07%** (2.29% + 5.33% + 4.45%) under 1-Sun illumination. This means that 87.9% of the absorbed light, which is ca. 77.5% of the power input, is used for vapor generation. As such, based on this analysis, 68.1% of the solar input was used to generate vapor. This value is slightly higher than the value of its solar-to-vapor conversion efficiency determined based on the solar evaporation rate of PPC20 which is found to be 63.2%. This indicates that the rate of vapor generation and removal from the surface of PPC20 is slightly lower than its photothermal conversion efficiency. This may be due to the presence of closed pores which are inefficient escape channels for the vapor generated.

PPC30

Conductive heat loss

Conductive heat flux (J_{cond}) from PPC30 (in the floating mode) to the bulk water can be determined based on Equation S1:

$$J_{cond} = Cm\Delta T_{bulk} = 30.24 \text{ J}$$

Where C is the specific heat capacity of water (4.2 kJ °C^{-1} kg^{-1}), m represents the weight of bulk water (3 g), and ΔT_{bulk} (21.2 – 18.8°C) represents the increased temperature of the bulk water after stable steam generation.

Energy input during solar evaporation, $E_{in} = I_{in} \times A \times t = 936$ J

Where A is the irradiated area of PPC10 (0.000064 m^2) and t is the time duration of the solar evaporation (3600 s).

Conductive heat loss = $J_{cond}/E_{in} \times 100\%$ = 30.24 J/936 J × 100% = **3.23%**

Radiative heat loss

Radiative heat transfer can be calculated using the Stefan-Boltzmann (Equation S2):

$$\Phi = \varepsilon A\sigma \, (T_{PPC30}^4 - T_{ambient}^4)$$

Whereby Φ denotes the heat flux, ε is the emissivity, A is the area of the evaporation surface (0.000064 m^2), σ is the Stefan-Boltzmann constant (5.67x10^{-8} W m^{-2} K^{-4}), T_{PPC30} and $T_{ambient}$ are the temperatures of PPC30 (299.9 K) and ambience (291 K), respectively. Since PPC has a relatively high light absorption, it can be treated as a black-body in thermal equilibrium with an ε of 1.

Radiation flux, $J_{rad} = \Phi/A = \varepsilon\sigma \, (T_{PPC30}^4 - T_{ambient}^4) = 52.1$ W m^{-2}

Radiative heat loss = $J_{rad}/I_{in} \times 100\%$ = 52.1 W m^{-2} /1000 W m^{-2} × 100% = **5.21%**

Convective heat loss

Convective heat transfer, P_{conv} can be calculated by the Newton's law of cooling (Equation S3):

$$P_{conv} = hA(T_{PPC30} - T_{ambient})$$

Where h is the convective heat transfer coefficient (5 W m^{-2} K^{-1}).

Convective flux, $J_{conv} = P_{conv}/A = h(T_{PPC30} - T_{ambient}) = 44.5$ W m^{-2}

Convective heat loss = $J_{conv}/I_{in} \times 100\%$ = 44.5 W m^{-2} /1000 W m^{-2} × 100% = **4.45%**

Therefore, the total heat loss of PPC30 (in the floating mode) is **12.89%** (3.23% + 5.21% + 4.45%) under 1-Sun illumination. This means that 87.1% of the absorbed light, which is ca. 79.7% of the power input, is used for vapor generation. As such, based on this analysis, 69.4% of the solar input was used to generate vapor. However, the value of solar-to-vapor conversion efficiency determined based on the solar evaporation of PPC 30 was found to be 56.3%. The fact that the heat utilization efficiency is higher than that of solar-to-vapor conversion efficiency may be due to inefficient vapor escape due to the predominance of closed pores in PPC30.

S2. Supplementary Figures

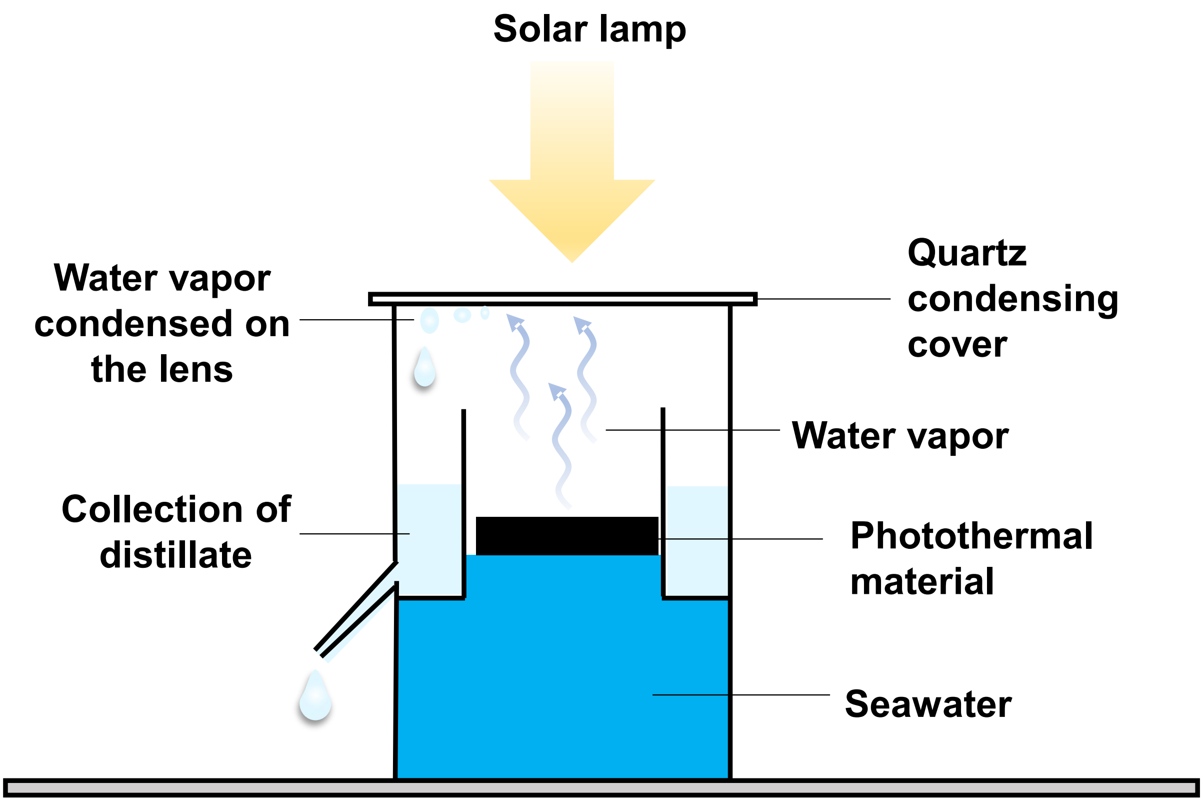

Figure S1. Schematic showing the condensation chamber used to collect the distillate.

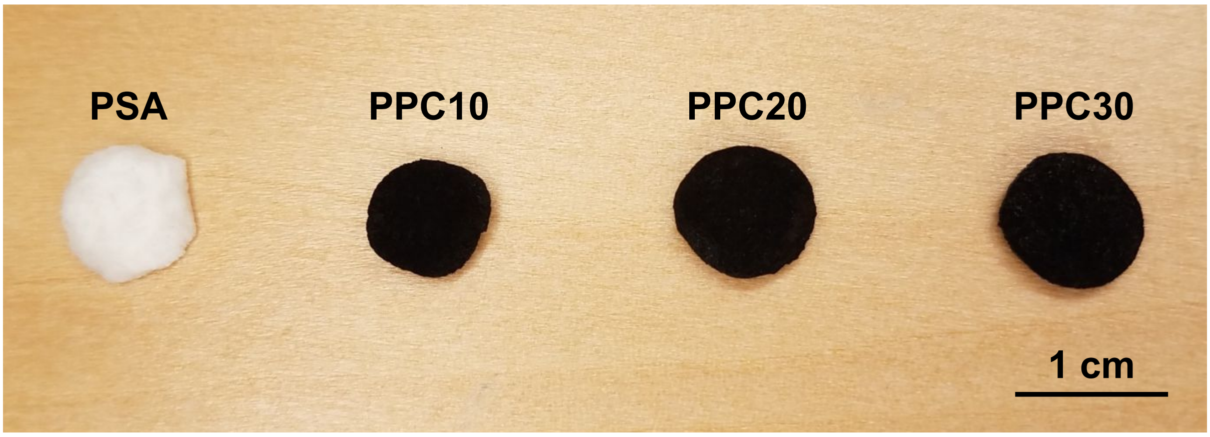

Figure S2. Digital photographs of the cryogel samples.

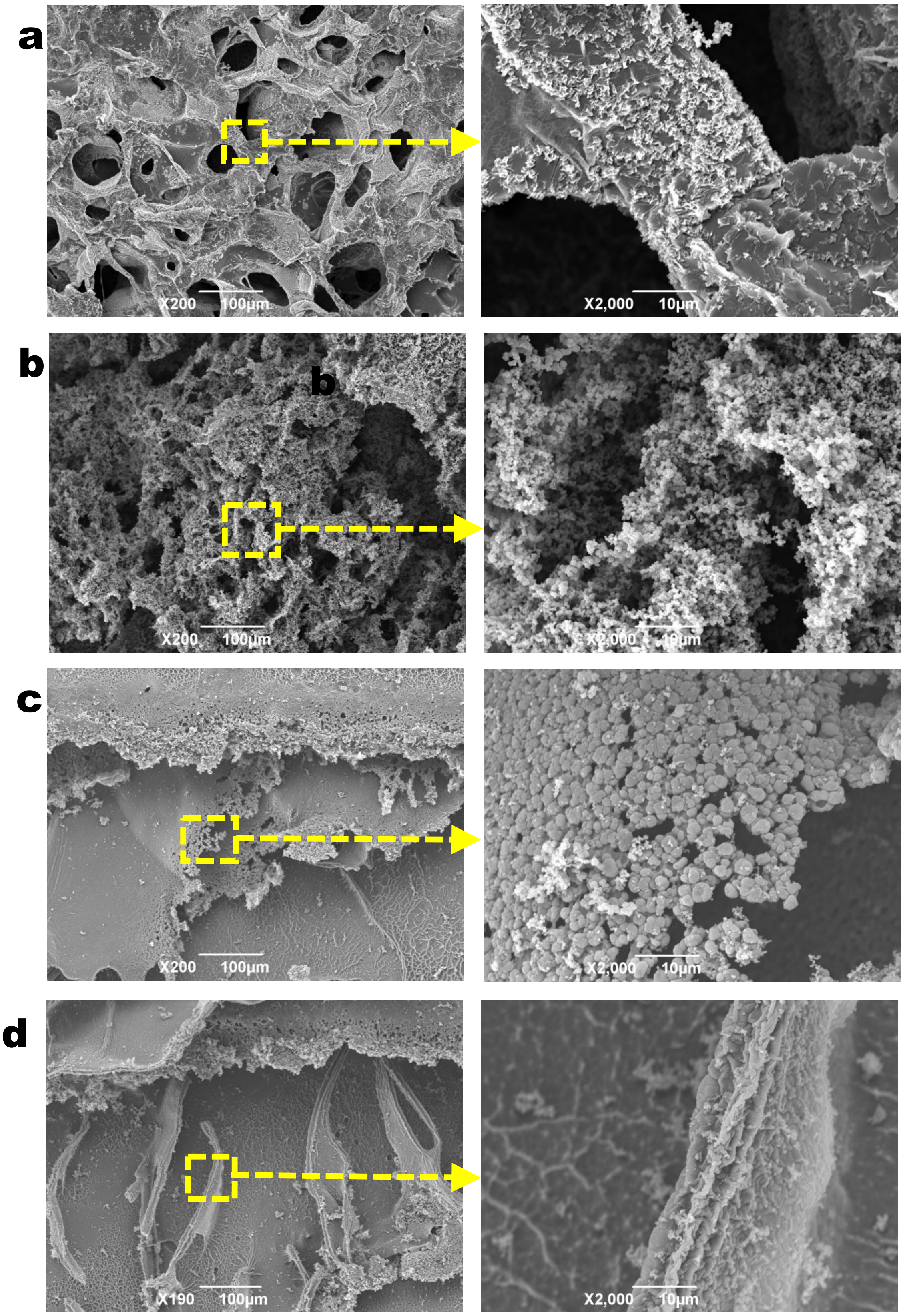

Figure S3. SEM showing the different morphologies in (a) PSA, (b) PPC10, (c) PPC20 and (d) PPC30.

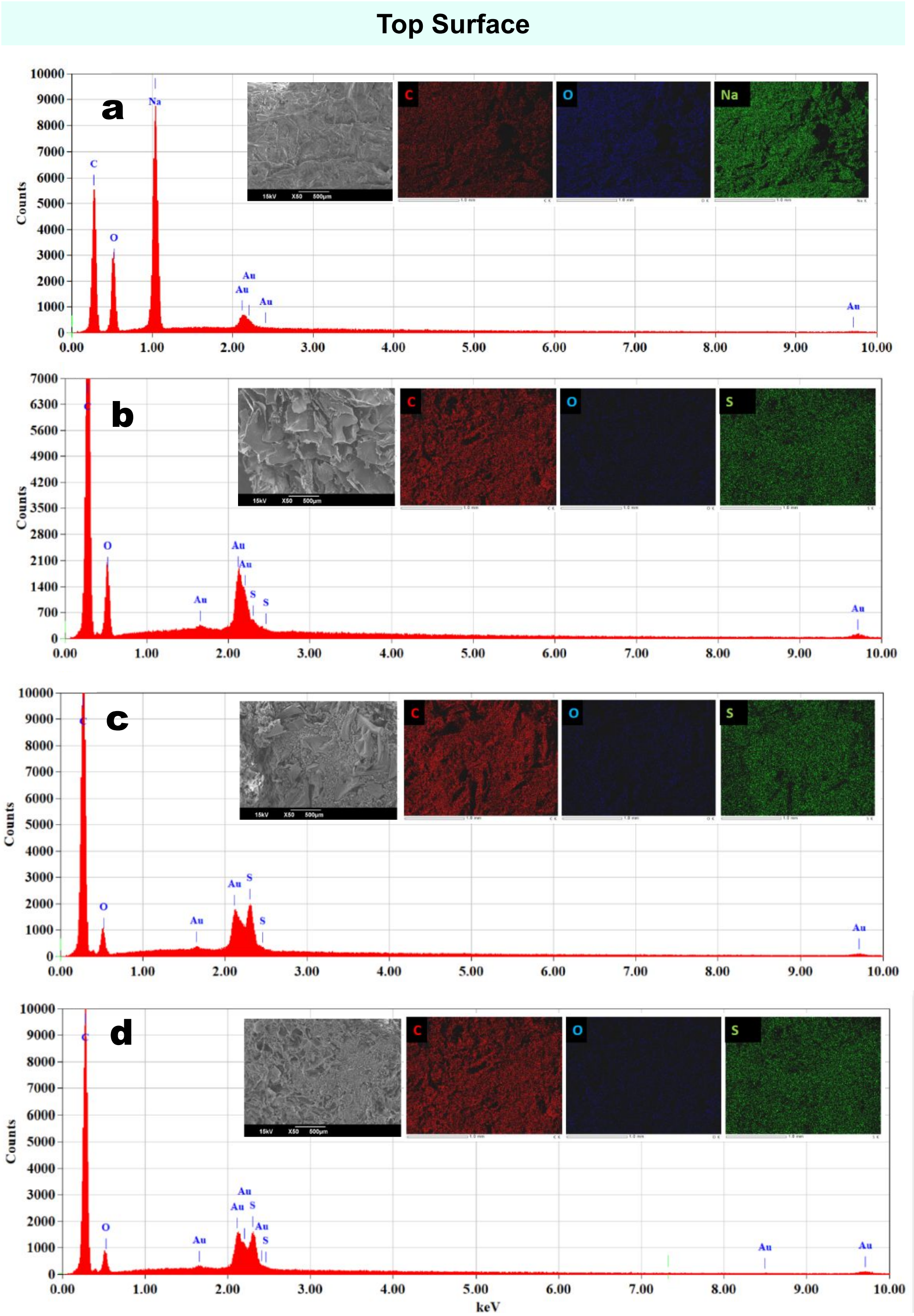

Figure S4. SEM-EDS elemental mapping for the top surface of (a) PSA, (b) PPC10, (c) PPC20 and (d) PPC30. Note that the Au peak is attributed to Au coating on the sample before the analysis.

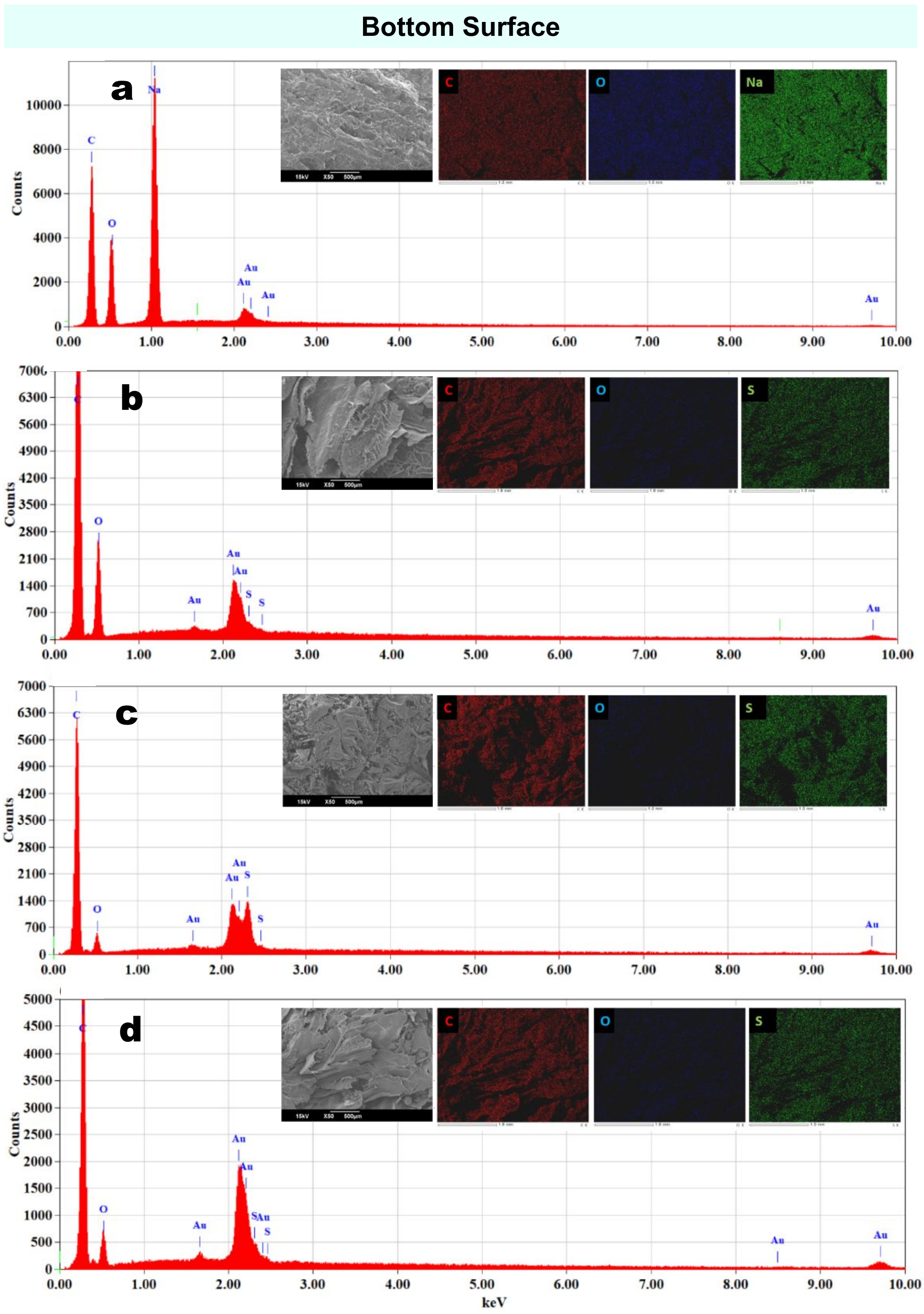

Figure S5. SEM-EDS elemental mapping for the bottom surface of (a) PSA, (b) PPC10, (c) PPC20 and (d) PPC30. Note that the Au peak is attributed to Au coating on the sample before the analysis.

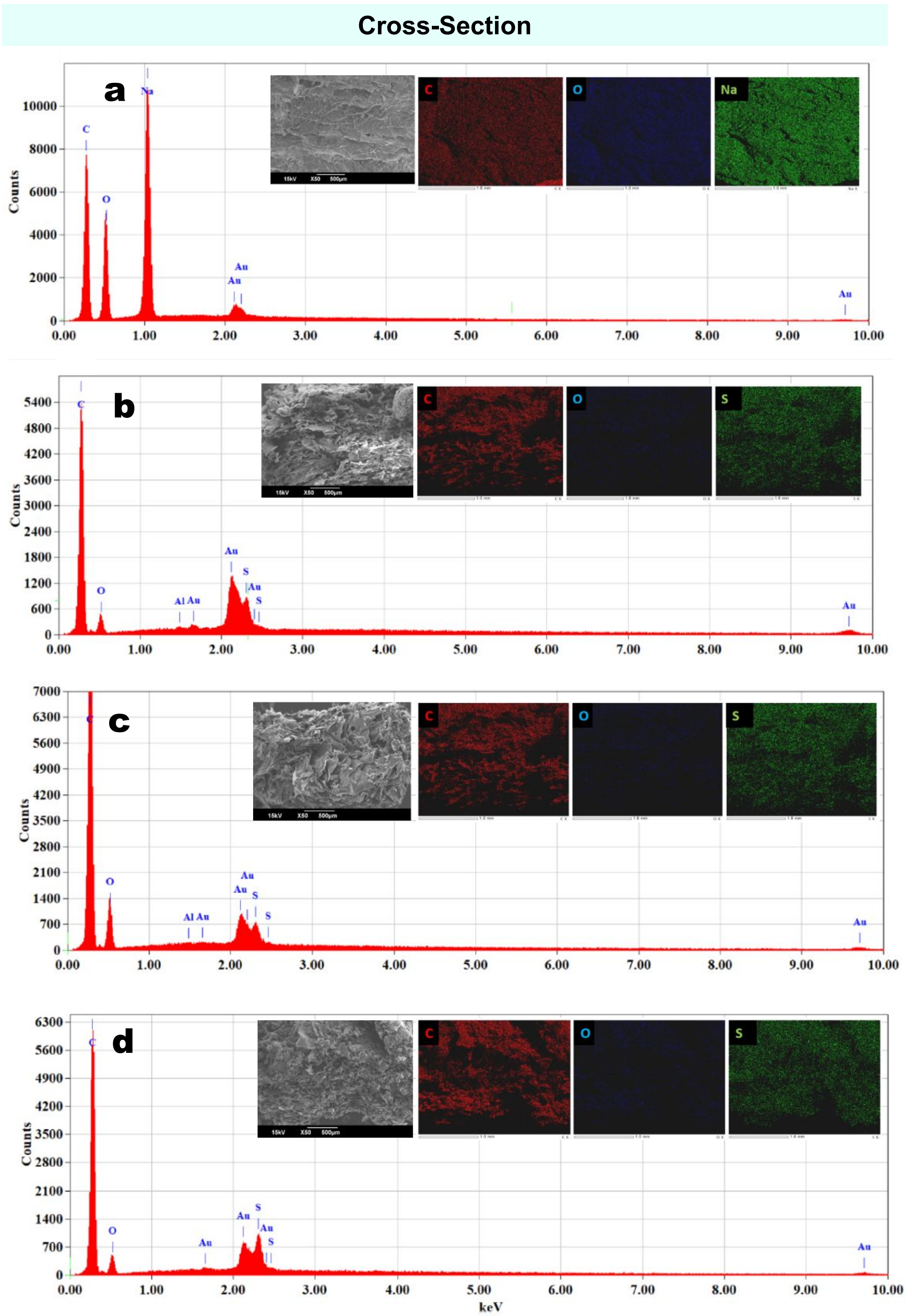

Figure S6. SEM-EDS elemental mapping for the cross-sectional surface of (a) PSA, (b) PPC10, (c) PPC20 and (d) PPC30. Note that the Au peak is attributed to Au coating on the sample before the analysis while the Al peak is due to possible contamination from the Al stubs.

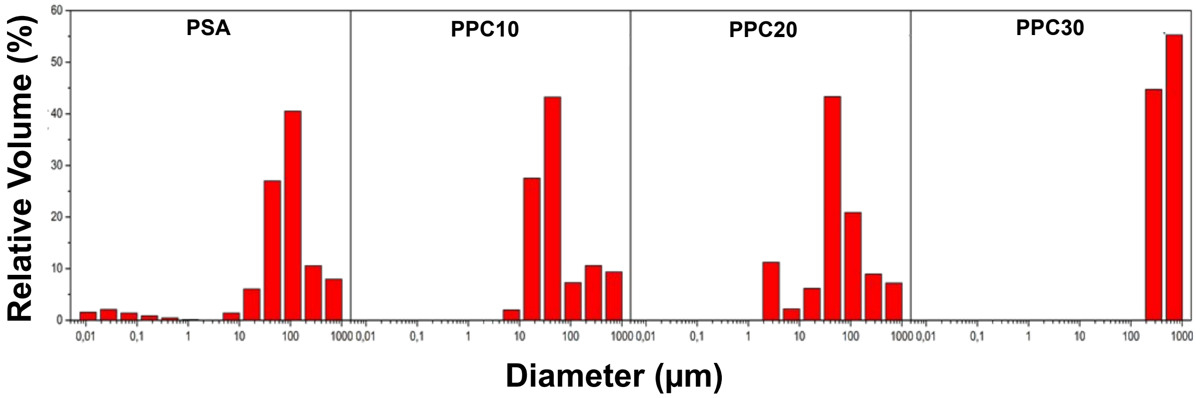

Figure S7. Pore-size distribution of the samples as determined from mercury intrusion porosimetry.

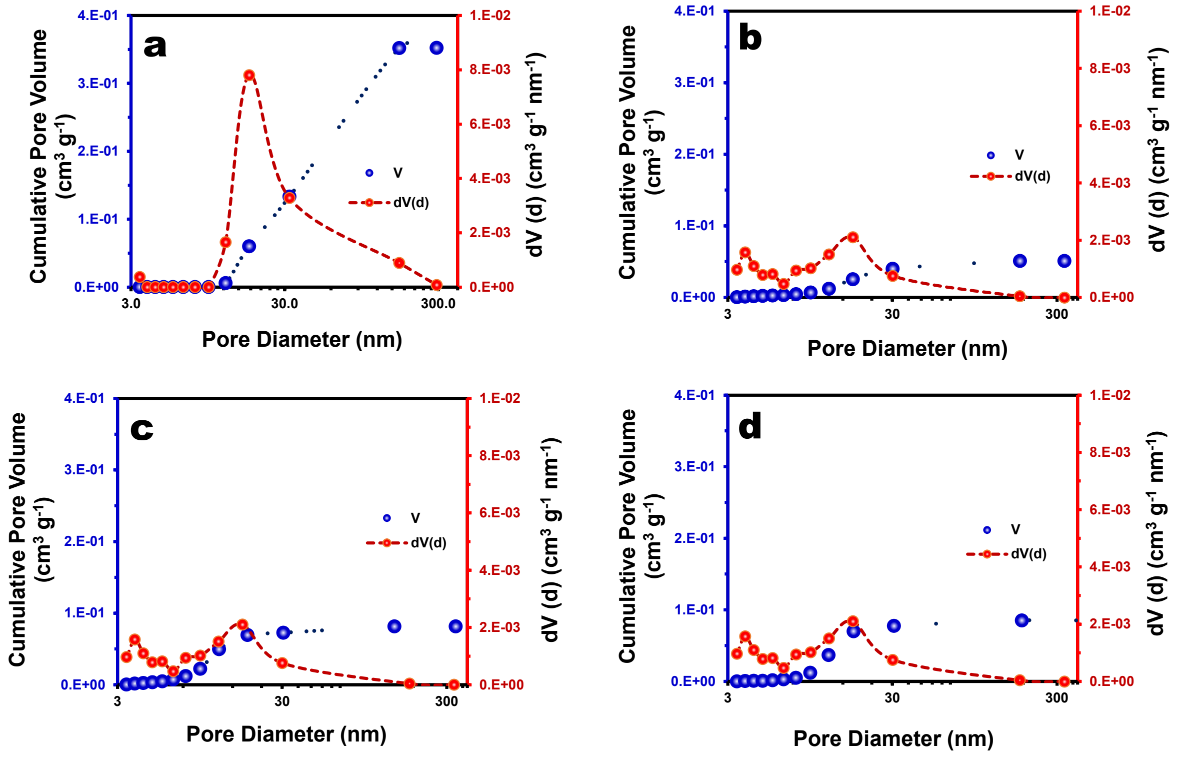

Figure S8. BJH pore-size distribution for (a) PSA, (b) PPC10, (c) PPC20 and (d) PPC30.

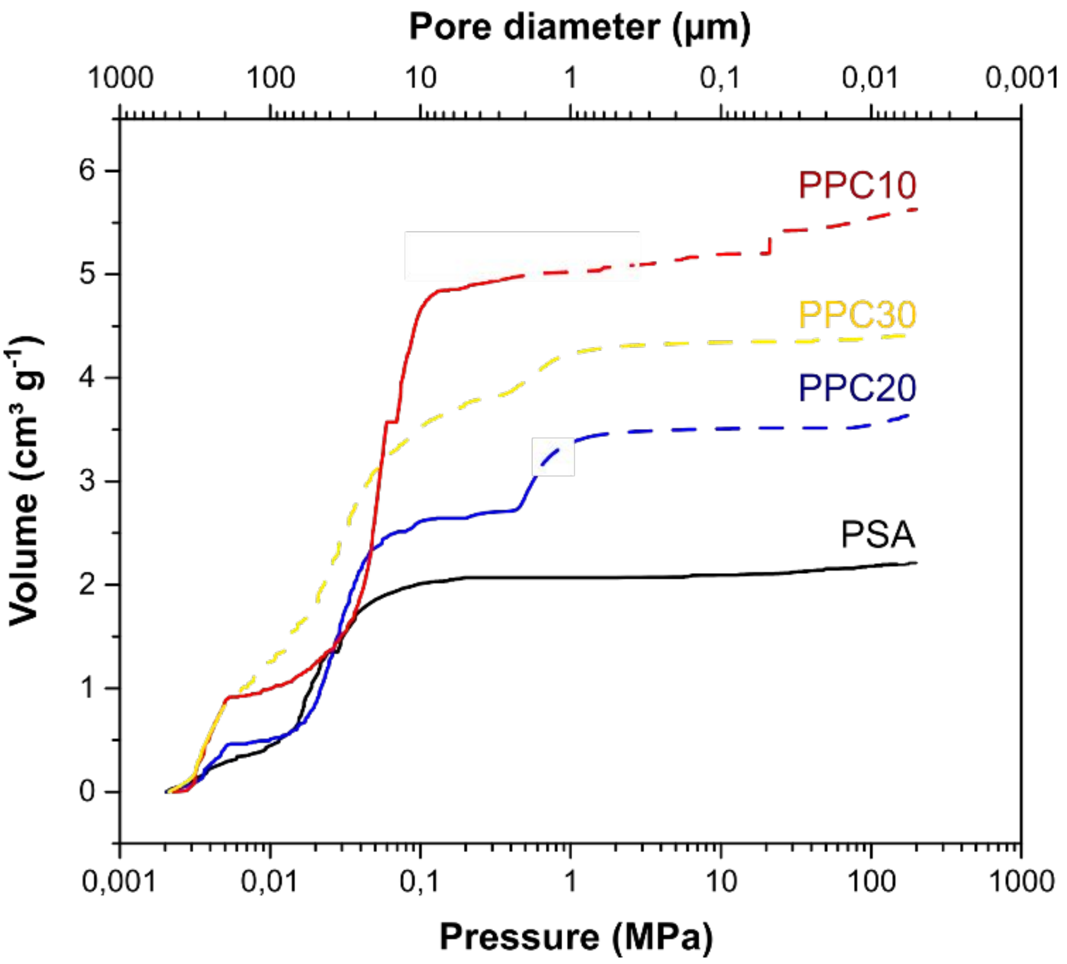

Figure S9. Mercury intrusion curves of the samples analyzed at a pressure range from 0.001 MPa to 200 MPa. Note that the dash lines represent the void volume of the closed pores opened during the analysis.

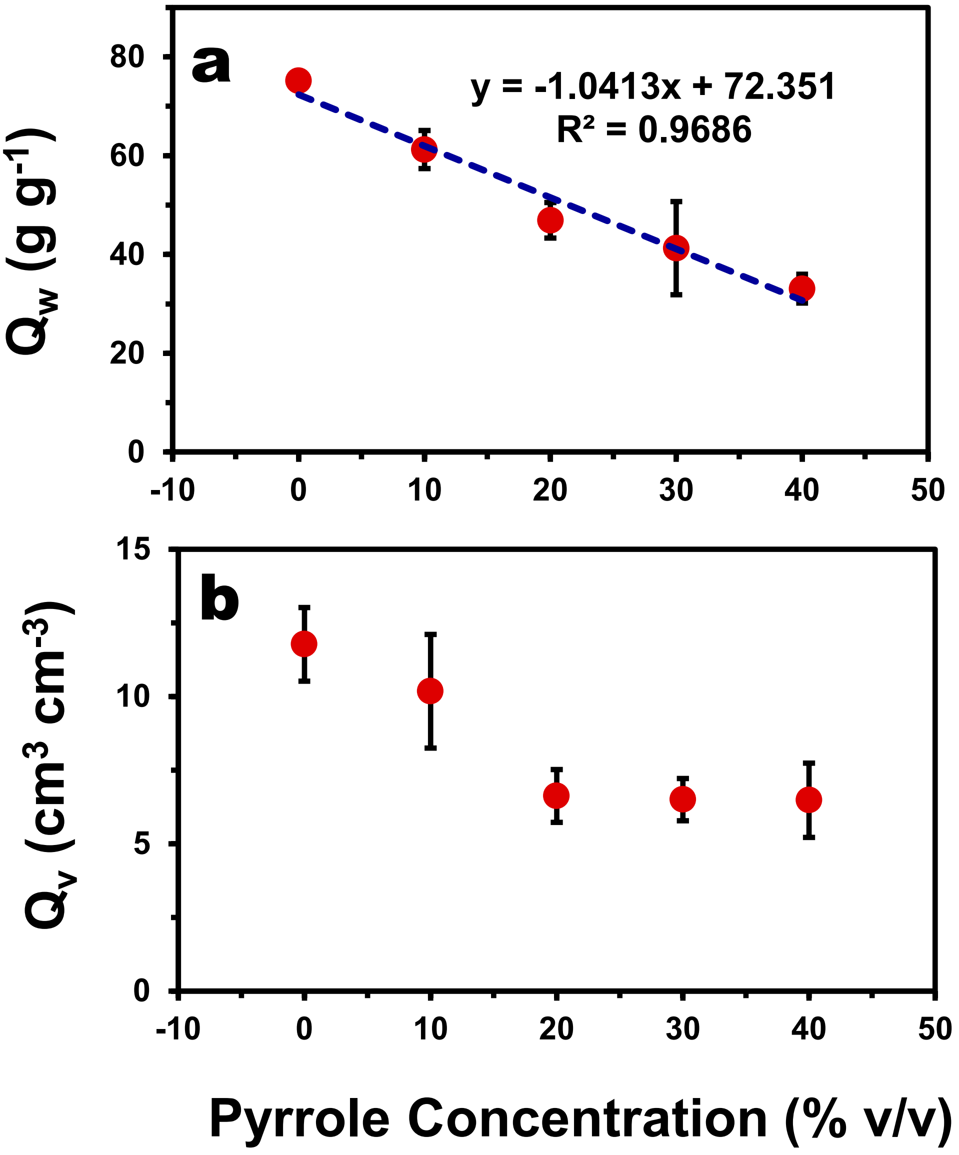

Figure S10. (a) Gravimetric and (b) volumetric swelling degrees of PSA cryogels in pyrrole solutions of varying concentrations.

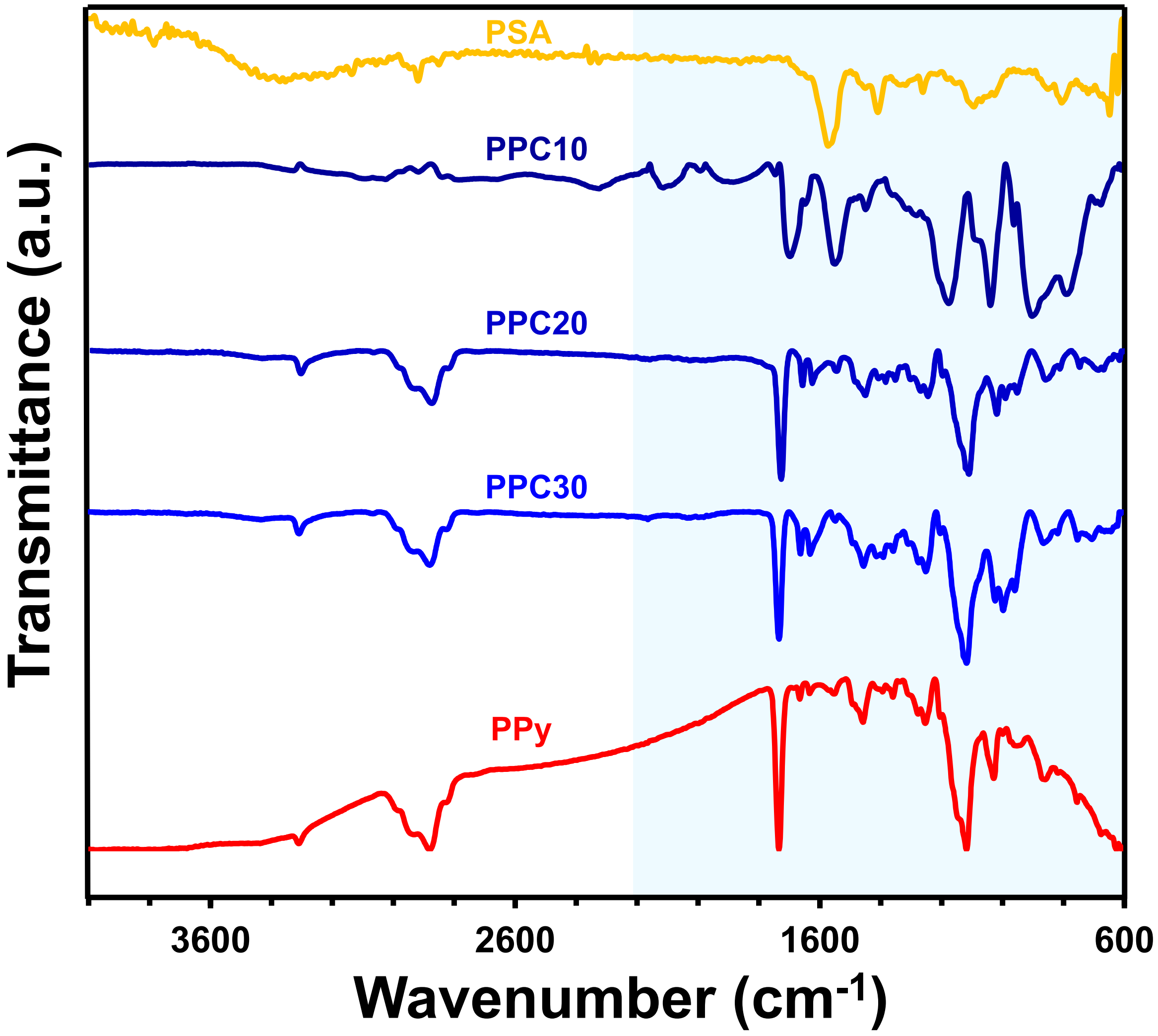

Figure S11. Wide-scan FITR spectra for all samples. Note that the fine details in the blue-shaded region are shown in Fig. 2 in the main paper.

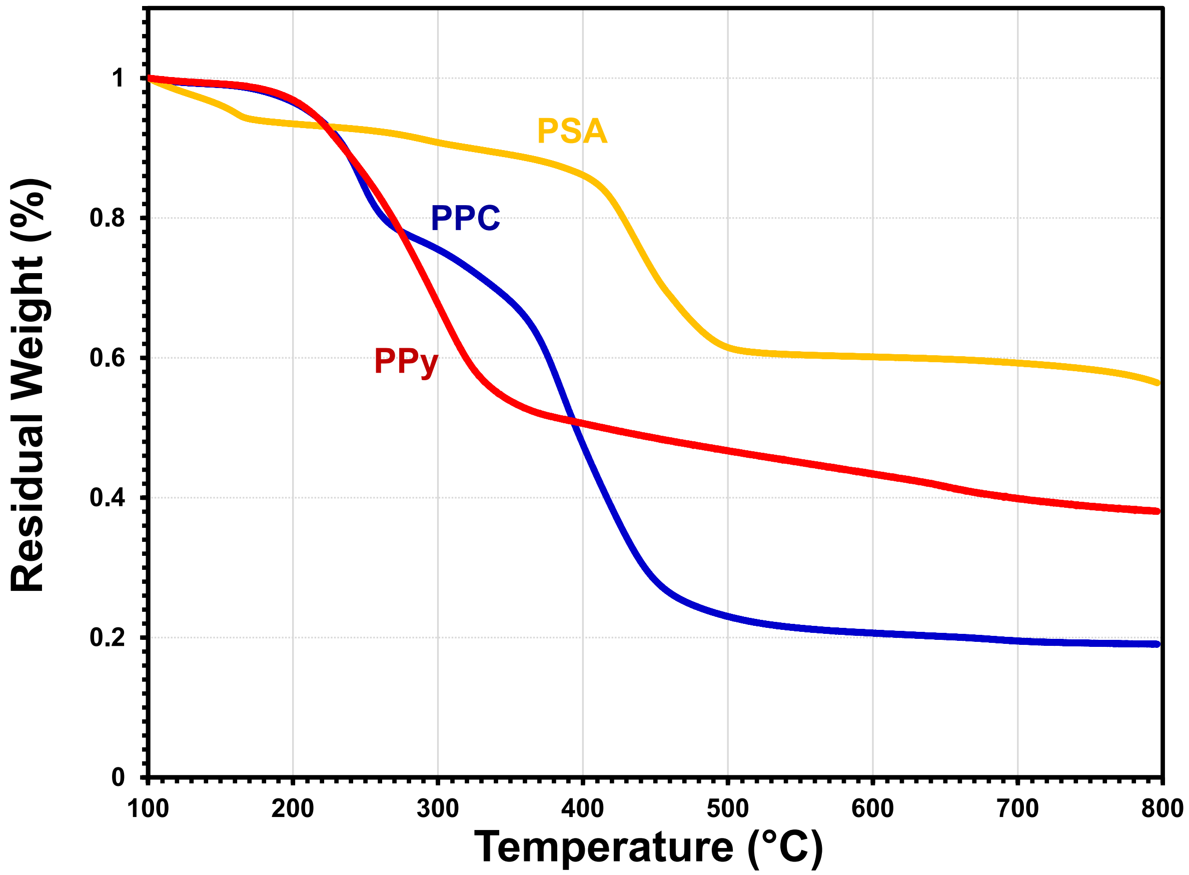

Figure S12. Thermogram showing the mass losses of PSA, PPy and PPC.

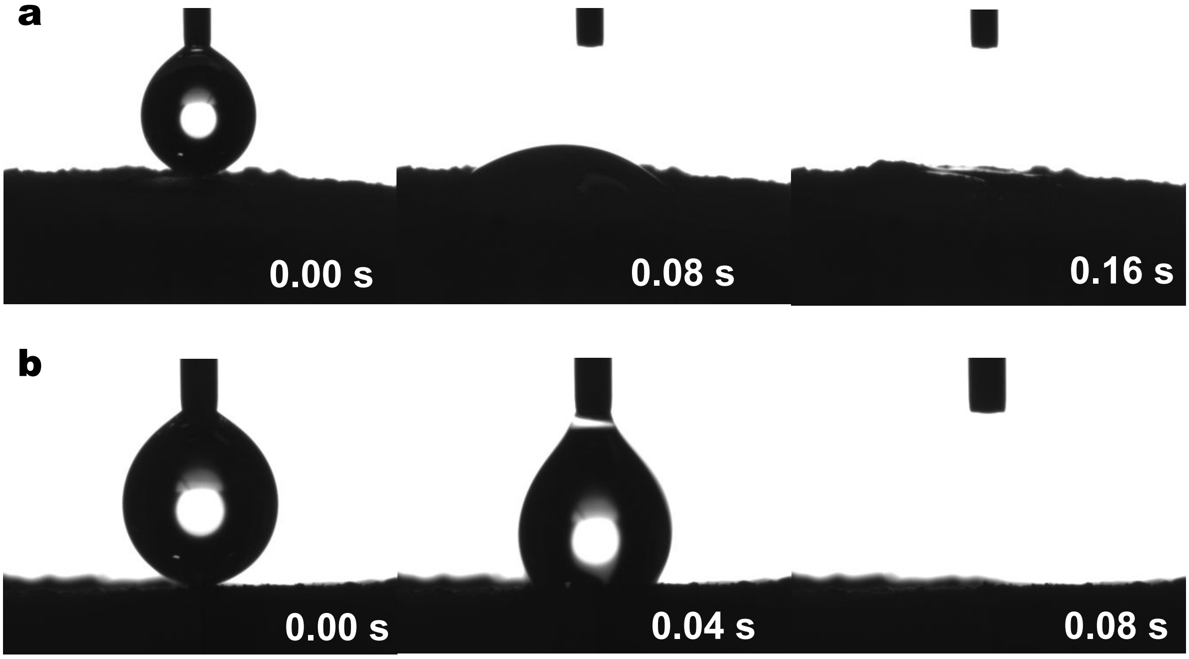

Figure S13. Video stills from contact angle analysis of (a) PSA and (b) PSA/PPy as represented by PPC30.

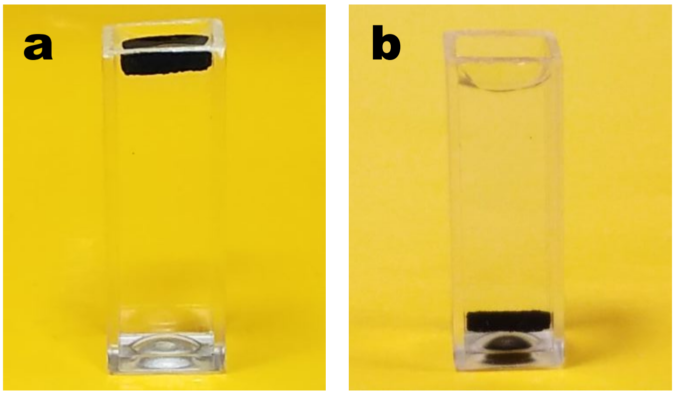

Figure S14. (a) PPC10 remains floating at the end of the 48-h solar evaporation test in synthetic seawater. (b) PPC10 sinks after 72-h immersion in synthetic seawater.

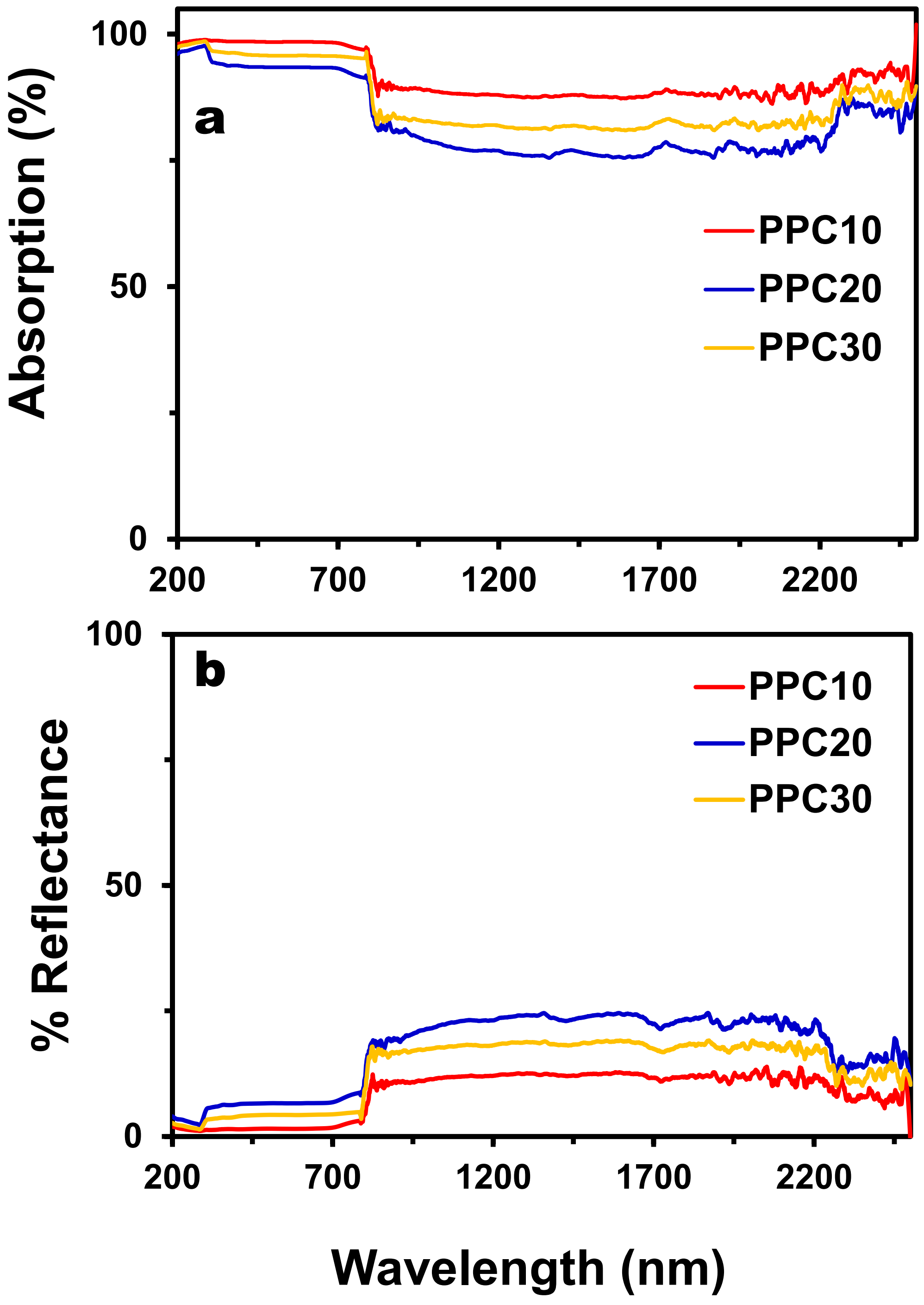

Figure S15. UV-vis-NIR (a) absorption and (b) reflectance spectra of the PPC samples.

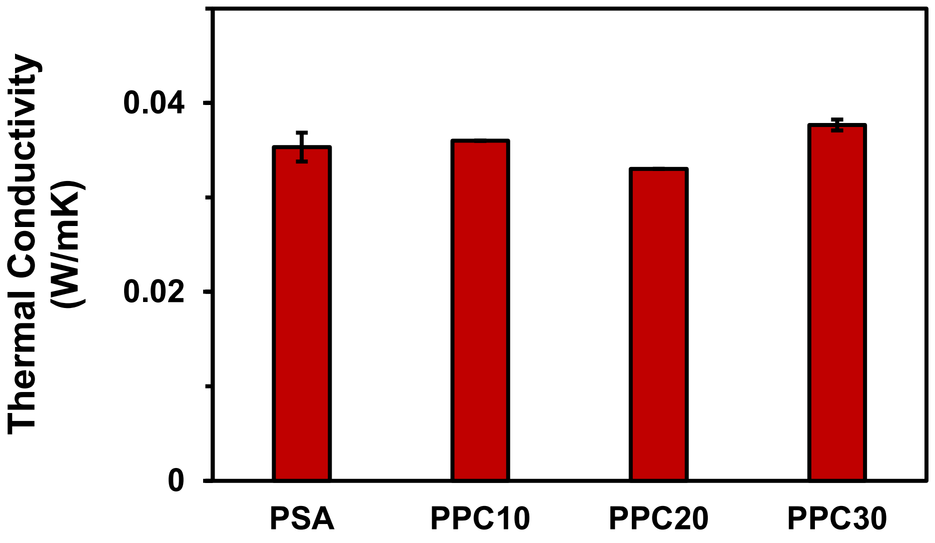

Figure S16. Thermal conductivity of the samples.

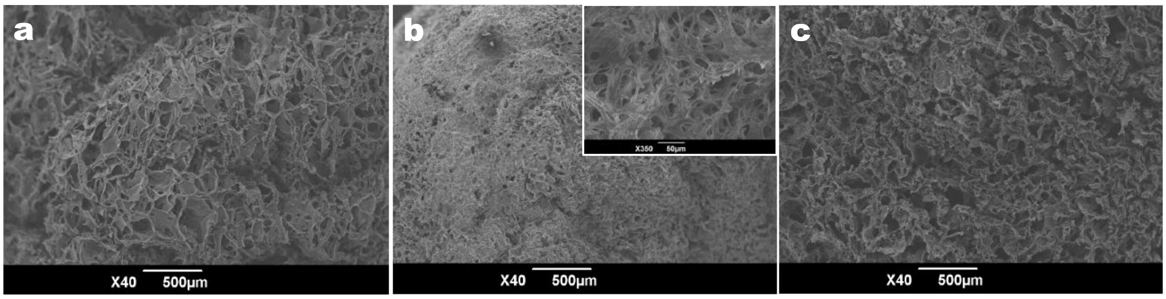

Figure S17. Morphology of (a) fresh PPC10, (b) PPC10 its use in solar evaporation of synthetic seawater for 48 h and (c) used PPC10 that has been washed via immersion in MilliQ water for 1 h.

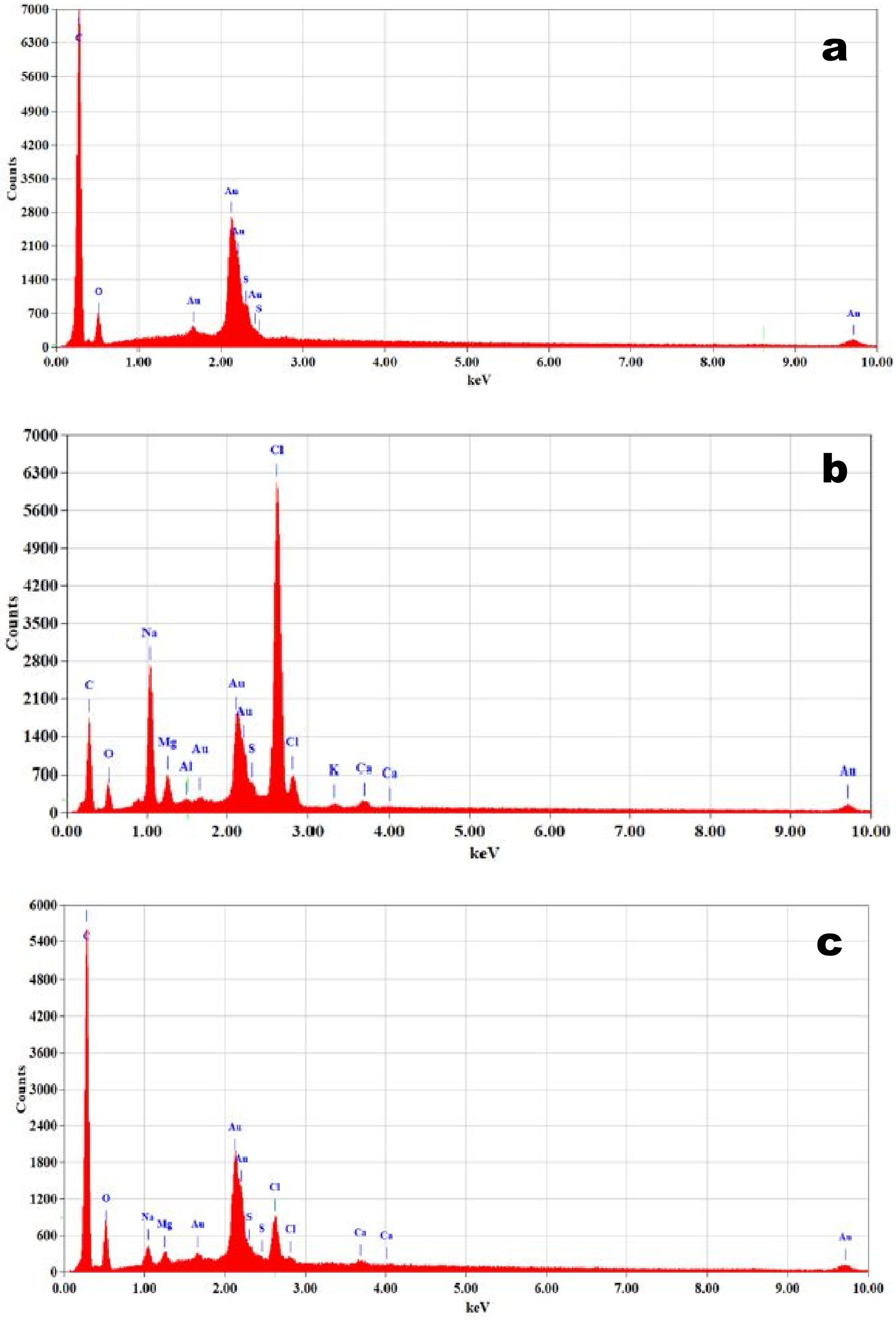

Figure S18. EDS spectra of the outer surface from (a) PPC10 before exposure to synthetic seawater, (b) PPC10 after the 48-h of continuous solar evaporation of synthetic seawater and (c) PPC10 after the 48-h of continuous solar evaporation of synthetic seawater followed by washing via immersion in MilliQ water for 1 h.

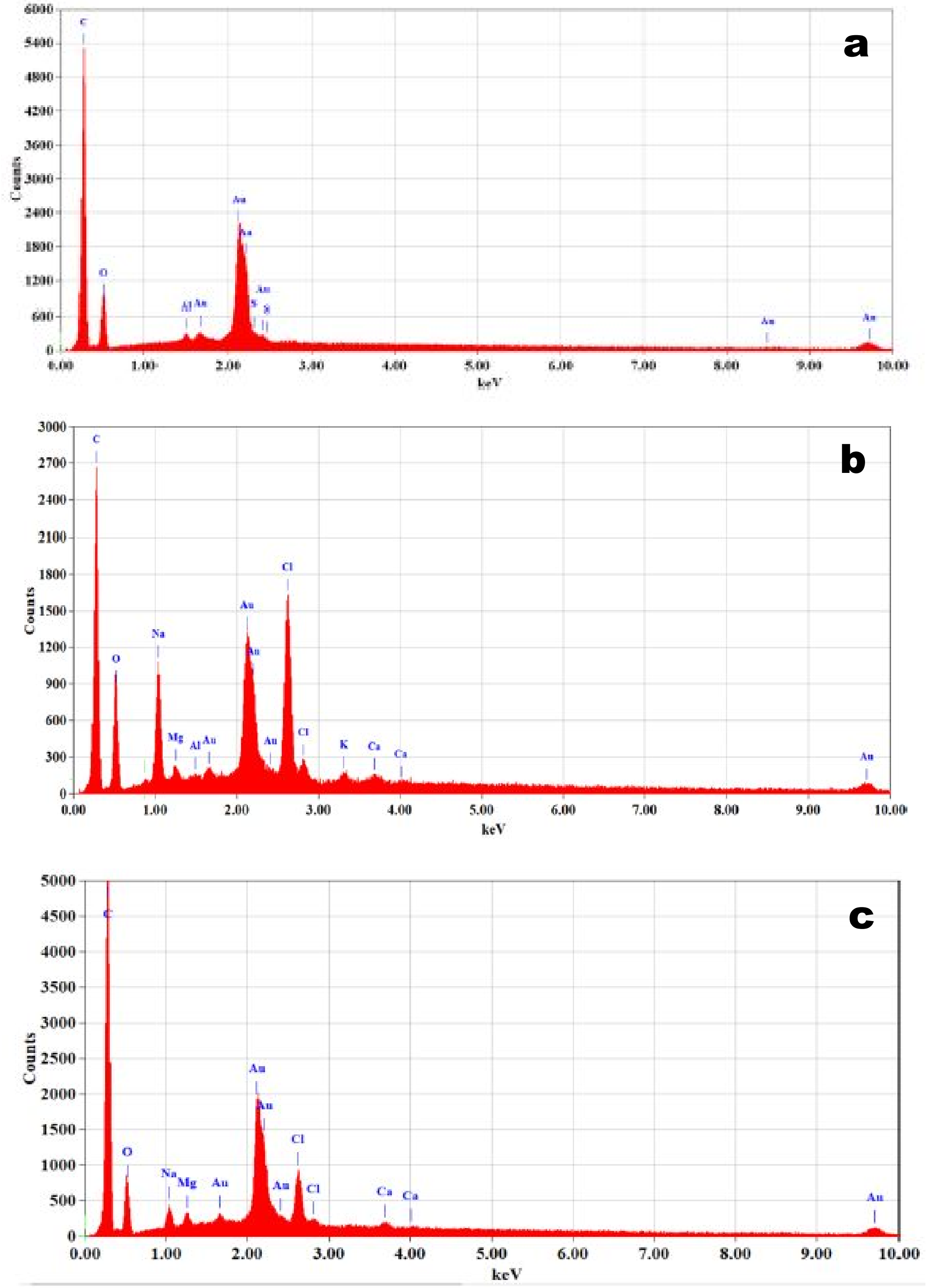

Figure S19. EDS spectra of the cross-sectional surface from (a) PPC10 before exposure to synthetic seawater, (b) PPC10 after the 48-h of continuous solar evaporation of synthetic seawater and (c) PPC10 after the 48-h of continuous solar evaporation of synthetic seawater followed by washing via immersion in MilliQ water for 1 h.

Table S1. Typical composition of the synthetic seawater

Components	Concentration (mg L^{-1})
Na^+	10700-11000
K^+	300-400
Ca^{2+}	400
Mg^{2+}	1320
Sr^{2+}	8.8
B (as H_3BO_3 in the solution)	5.6
Cl^-	19000-20000
CO_3^{2-}	140-200
SO_4^{2-}	2660
